# Intermittent Fasting on Neurologic Diseases: Potential Role of Gut Microbiota

**DOI:** 10.3390/nu15234915

**Published:** 2023-11-24

**Authors:** Mingke Guo, Xuan Wang, Yujuan Li, Ailin Luo, Yilin Zhao, Xiaoxiao Luo, Shiyong Li

**Affiliations:** 1 Hubei Key Laboratory of Geriatric Anesthesia and Perioperative Brain Health, Department of Anesthesiology, Wuhan Clinical Research Center for Geriatric Anesthesia, Tongji Hospital, Tongji Medical College, Huazhong University of Science and Technology, Wuhan 430030, China; m202176229@hust.edu.cn (M.G.); d202182020@hust.edu.cn (X.W.); yujuanli@hust.edu.cn (Y.L.); alluo@tjh.tjmu.edu.cn (A.L.); yilinzhao@hust.edu.cn (Y.Z.); 2 Department of Oncology, Tongji Hospital, Tongji Medical College, Huazhong University of Science and Technology, Wuhan 430030, China

**Keywords:** intermittent fasting, gut microbiota, neurodegenerative disease, neurologic disease

## Abstract

As the global population ages, the prevalence of neurodegenerative diseases is surging. These disorders have a multifaceted pathogenesis, entwined with genetic and environmental factors. Emerging research underscores the profound influence of diet on the development and progression of health conditions. Intermittent fasting (IF), a dietary pattern that is increasingly embraced and recommended, has demonstrated potential in improving neurophysiological functions and mitigating pathological injuries with few adverse effects. Although the precise mechanisms of IF’s beneficial impact are not yet completely understood, gut microbiota and their metabolites are believed to be pivotal in mediating these effects. This review endeavors to thoroughly examine current studies on the shifts in gut microbiota and metabolite profiles prompted by IF, and their possible consequences for neural health. It also highlights the significance of dietary strategies as a clinical consideration for those with neurological conditions.

## 1. Introduction

In recent decades, notable shifts have taken place in human dietary patterns. There has been a trend away from the typical breakfast-lunch-dinner pattern towards the adoption of unhealthy eating habits, including the consumption of frequent snacks and late-night snacking [1]. The irregular diet was found associated with development of diseases in nervous system [2]. Nutrition is a significant determinant in an individual’s overall lifestyle. Extensive findings indicate that certain nutrients or food constituents are potential cognitive enhancers [3]. Modifying dietary patterns, including alterations in meal timing and frequency, could enhance overall life quality and metabolic functioning, hence reducing the risk of developing metabolic syndrome and neurologic disorders [4].

Intermittent fasting (IF), with its various forms such as alternate-day fasting (ADF), periodic fasting (PF), time-restricted feeding (TRF), and the fasting-mimicking diet (FMD), offers the advantage of reduced caloric intake [5]. IF is economically feasible and has gained considerable acceptance compared to other dietary interventions [6]. A substantial body of research has consistently highlighted the positive effects of this dietary approach on multiple physiological systems within the human body. However, the specific influence of IF on the neurological system warrants further exploration [7]. The investigation into the impact of gut microbiota on brain function has emerged as a compelling area of research in recent years. Current scientific insights suggest that microbial communities could significantly influence the development and progression of various neurological conditions, such as Alzheimer’s disease (AD), Parkinson’s disease (PD), autism spectrum disorder (ASD), multiple sclerosis (MS), and stroke [8,9]. The proposed interaction between the gastrointestinal tract (GIT) and the central nervous system (CNS) is thought to be mediated by a variety of microbial metabolites produced in the gut.

Human GIT is home to a vast consortium of microorganisms, including bacteria, viruses, fungi, and others. Numerous studies have shown that gut bacteria can impact the host’s physiological and pathological states in the context of health and disease [10]. Conversely, the composition of the gut microbiota can be influenced by various factors, such as the host’s genetic makeup, lifestyle, diet, antibiotic use, and other relevant variables. The influence of gut microbiota on the CNS and the enteric nervous system (ENS) holds significant relevance concerning central nervous inflammation, neural development, and the modulation of mood and behavior [11]. Disruption in the equilibrium of intestinal microbes has been linked to an increased risk of various CNS disorders. An accumulating body of evidence suggests a bidirectional communication network between the CNS and intestinal bacteria, commonly referred to as the brain–gut axis [12].

This review seeks to scrutinize the compositional and functional changes in the intestinal microbiota, assess the evidence relevant to the risk of neurological disease, and advocate for IF as a viable and advantageous lifestyle interventions.

## 2. Intermittent Fasting (IF)

### 2.1. Concept and Modes

**Figure 1:**
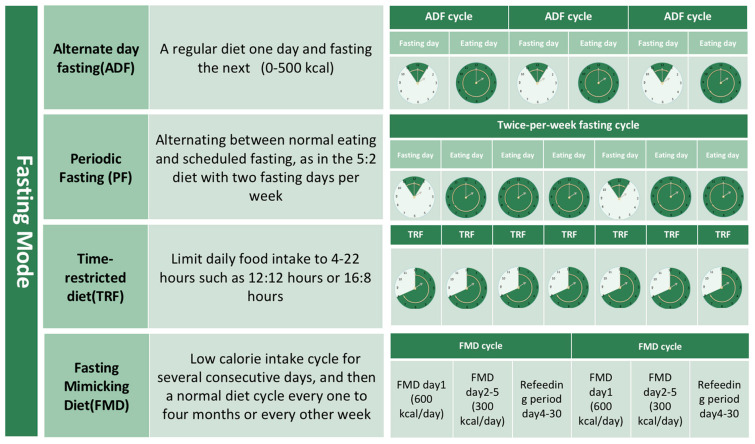
Types of IF. Four different types of IF were presented, including alternate day fasting (ADF), periodic fasting (PF), time-restricted feeding (TRF), and the fasting mimicking diet (FMD). Charts were used to visually represent the duration of fasting and the schedule of therapy for each IF approach. Intensified color is used to distinguish the eating periods inside the fasting regimen.

IF refers to a dietary regimen that involves cyclically alternating times of consuming food and abstaining from eating [5]. Compared to the traditional caloric restriction method, it imposes fewer limitations on dietary choices and primarily emphasizes adherence to certain eating windows rather than specific food choices. Currently, extant research has indicated that IF has demonstrated efficacy in facilitating weight loss [13], enhancing blood glucose and blood lipids, as well as immune regulation [14]. The practice of IF encompasses various commonly employed methods, as depicted in Figure 1.

### 2.2. The Extensive Benefits of IF

The beneficial effect of IF on important organ, such as cardiovascular system, brain, has been extensively investigated and elaborately summarized in recently published review papers [7,15,16,17,18]. Therefore, in the present review, we did not repeated this topic.

### 2.3. Potential Harm of IF

Although IF brings some of the aforementioned benefits, its safety has yet to be verified. The potential for the diet to elicit gastrointestinal symptoms, energy imbalance, eating disorders, and hormone problems remains uncertain. Each individual’s response to IF varies widely based on their unique health status, genetics, and lifestyle. Therefore, while IF offers benefits, it requires careful consideration and medical guidance for those with specific health concerns.

#### 2.3.1. Diet Quality and Energy Imbalance

There is a prevalent hypothesis suggesting that the intake of high-calorie food may potentially rise during the designated eating period within the practice of IF [19]. One studies found there were no statistically significant differences in the consumption of sugar, saturated fat, cholesterol, and sodium when comparing the experimental group to the control group. However, the consumption of dietary fiber in the experimental group was significantly lower compared to that in the control group. Although the finding from this study indicated that IF did not significantly impact the overall dietary quality, it suggested IF increased the overall consumption of dietary fiber [20,21], as it has been shown to promote the gastrointestinal well-being of individuals [20].

#### 2.3.2. Hormone Disorders

Fasting may alter thyroid hormone secretion and insulin signaling [22]. Fasting affects thyroid hormone levels in healthy individuals and those with subclinical hypothyroidism [22]. In patients with obesity or subclinical hypothyroidism, alternate-day fasting (ADF) and continuous calorie restriction (CR) did not induce substantial changes in circulating fT4, fT3, and TSH [23]. IF reduced T3 levels slightly in people with lower body weight, but not in those with obesity and subclinical hypothyroidism [24]. In premenopausal women, a 24-week 5:2 IF regimen did not significantly modify testosterone, androstenedione, dehydroepiandrosterone sulfate, sex hormone binding globulin (SHBG), or prolactin levels [25]. In postmenopausal women, TRE did not lead to changes of estrogen, progesterone, androgen, and SHBG but induced DHEA decrease [26]. Recent studies have pointed out that maternal long-term IF before pregnancy would destroy the intestinal barrier of offspring by inhibiting beneficial microbiota (such as Lactobacillus integrans), resulting in dysfunction of glucose and lipid metabolism in offspring [27]. As to IF in children, it is still under debate and more evidence is needed to its safety.

#### 2.3.3. Dietary Patterns and Mental Health

IF possibly impacts dietary habits and mental health with detrimental consequences. Its emphasis on strict timing and food restriction could be harmful to those with a history of eating disorders, fostering an unhealthy relationship with food focused more on fasting than on balanced nutrition [28]. This pattern led to nutrient deficiencies and disrupt natural hunger and satiety cues, causing overeating during permitted periods or ignoring hunger signals, thereby exacerbating unhealthy eating behaviors and potentially causing metabolic and gastrointestinal problems. Moreover, the mental health effects of IF may be substantial. It exacerbated symptoms in individuals with mood disorders such as depression and anxiety through added stress and hormonal changes. Those with bipolar disorder may experience mood destabilization due to changes in diet and routine [29], and individuals with obsessive-compulsive disorder might find their food-related compulsions and rituals worsened [30]. The strict regimen of IF increased stress and anxiety, leading to feelings of social isolation, and strain relationships due to conflicts with social eating customs. This heightened focus on food and body image necessitates a careful, balanced approach to IF, especially for those with existing mental health issues.

#### 2.3.4. Other Side Effect of IF

Although a recent review outlined that IF would be an emerging dietary intervention for autoimmune diseases [31], but another team argued that long-term fasting could result in monocytes re-enter the bone marrow and reduced the host response to infection [32]. Some studies reported IF might induce constipation and headache, especially at the early period of IF [18]. Generally, its side effects are mild, but that does not mean we can neglect it.

## 3. The Role of Intestinal Microflora in IF-Induced Improvement in Cognitive Protective Effect

**Figure 2:**
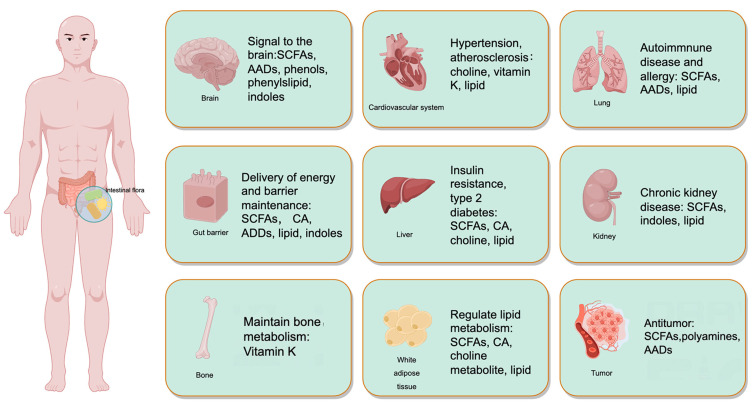
Impact of gut microbiota-derived metabolites on host organ systems. This figure illustrates the diverse physiological and pathological effects of metabolites produced by the gut microbiota on multiple host organ systems. Specifically, the metabolites have the potential to influence the brain, cardiovascular system, lungs, liver, kidneys, intestinal barrier, bone, adipose tissue, and cancerous tissues. AADs—amino acid derivatives; CA—cholic acids; SCFAs—short-chain fatty acids.

The benefits of IF on neuroplasticity and cognitive function have been extensively examined and reviewed in recent literature [7]. Figure 2 illustrates the impact of gut microbiota on the physiological and pathological processes of the host’s organs. This review delves into the mechanisms underlying IF-induced improvements in brain health and disease.

Among the various potential mechanism, the gut microbiota has attracted increasing attention for its role in influencing IF’s effects on the body’s physiological activities, including brain function [33]. We collated available studies that examined the influence of IF on the composition and metabolites of gut microbiota from clinical trials and preclinical models in Table 1 and Table 2. Additionally, we explored in detail the progress made in understanding how IF impacts brain function through the modulation of intestinal microflora.

### 3.1. The Changes of Microbial Metabolites Following IF

The bioactive metabolites produced by bacteria residing in the intestinal tract are capable to influence the host organism’s physiological processes, either through direct or indirect pathways [68]. The effect of gut microbiota on host physiology has been primarily attributed to microbial metabolites. Humans consume three primary macronutrients—carbohydrates, proteins, and fats—in their daily diet [69]. These macronutrients are not completely digested by the body. Undigested nutrients can bypass early digestion and serve as substrates for intestinal microbial metabolism, leading to the production of microbial metabolites such as short-chain fatty acids (SCFAs), amino acids, bile acids, and vitamins, which influence host physiological functions [70]. Table 3 provides a comprehensive overview of the systemic impacts of gut microbiota-generated metabolites on the host organism and examines the effects of various metabolites on metabolic disorders affecting the nervous system.

#### 3.1.1. Short-Chain Fatty Acids (SCFAs)

SCFAs are primarily synthesized by anaerobic bacteria in the colon through the fermentation of dietary fiber and resistant starch and include acetic acid, propionic acid, and butyric acid [83,84]. They not only serve as an energy source within the gut but also play a crucial role in modulating the host’s biological responses, influencing multiple body systems [83,84,85,86]. The quantity of SCFAs in the intestinal mucosa and feces of persons with a diagnosis of inflammatory bowel disease exhibit a substantial reduction in comparison to those observed in the healthy control group [87]. The incidence of colorectal cancer is also linked to the decrease in gut microbiota and SCFAs [88]. Individuals who are overweight experience weight loss following prolonged administration of propionate [89]. Simultaneously, it has been noted by researchers that SCFAs have the capability to mitigate insulin resistance through the prohibition of histone deacetylases [90]. Studies suggest SCFAs can stimulate the production of glucagon-like peptide-1 (GLP-1) and peptide YY, affecting both endocrine and nervous systems [91]. One of the processes through which intestinal microbes regulate blood pressure is through SCFAs, which interact with two receptors known as GPR41 and Olfr78. The Olfr78 knockout mice for exhibit a state of hypotension, whereas the knockout mice for GPR41 display a state of hypertension [92]. The conception of the gut–brain axis suggests that modulating microbes and their metabolites can be a viable strategy for enhancing and mitigating neurodegenerative disorder [93]. This transportation is facilitated by the mono-carboxylic acid transporter [94,95]. Moreover, it has been demonstrated that various IF protocols can result in elevated concentration levels of SCFAs within the body, as well as alterations in the microbial populations responsible for SCFA production (e.g., Bacteroides, Clostridium, and Rumen Cocci). The results of this study indicate that IF might regulate the composition of SCFAs in the gut environment by impacting the variety and quantity of microbial populations.

#### 3.1.2. Amino Acids and Derivatives

Intestinal bacteria possess the ability to encode and synthesize a diverse range of amino acids. Furthermore, the intestinal microbiota also engages in the modification of dietary amino acids by processes of deamination and decarboxylation. This includes the metabolic conversion of aromatic amino acids (such as tyrosine, alanine, and tryptophan) into a range of subsequent products [96,97]. Tyrosine undergoes metabolic processes resulting in the production of tyramine, which subsequently gives rise to two distinct types of catecholamines, namely dopamine and norepinephrine. As an illustration, the amino acid tyrosine has the capacity to undergo metabolic processes resulting in the formation of 4-ethylphenol. Subsequently, the host organism efficiently sulfates 4-ethylphenol to produce 4-ethylphenyl sulfate (4EPS). In the mouse model exhibiting characteristics of autism and schizophrenia, as well as in the sample of children diagnosed with autism, there is an observed rise in the occurrence of 4EPS [98]. The process of tryptophan decomposition results in the formation of indole derivatives, tryptamine, and kynurenine. Indole has been shown to enhance the production of connexin, hence reinforcing the integrity of the intestinal barrier function [99]. Additionally, indole has been found to stimulate the release of GLP-1 [100]. It is known that the application of GLP-1 receptor agonists can act on the central nervous system, which can inhibit appetite, increase satiety, and reduce caloric intake [101]. The further investigation on the central nervous system of GLP-1 aiming not on appetite has broad prospects. The impact of kynurenine metabolites on neuronal glutamate receptors has been observed to influence cognitive processes, as well as behaviors associated with worry and stress [102,103]. Glutamate, an excitatory neurotransmitter, can undergo metabolic conversion by the enzyme glutamate decarboxylase, resulting in the production of gamma-aminobutyric acid (GABA). Several researches have indicated that the bacterial strain accountable for the synthesis of GABA has promise in alleviating depression symptoms and anxiety-related behaviors in mice. The potential association between aberrations in the glutamate/GABA system within the brain and many disorders such as anxiety, depression, schizophrenia, and other pathological conditions has been postulated. The dysregulation of the dopamine system has been found to be associated with mood disorders, such as depression, as well as AD [104,105,106]. Dopamine has been used as a preclinical intervention for the management of depression, anxiety, and cognitive dysfunction. Further investigation is warranted to examine the alterations in neurophysiological function in hosts resulting from the metabolic conversion of amino acids into various compounds by gut microbes.

#### 3.1.3. Bile Acid

Bile acid is a byproduct of the metabolic process of cholesterol and serves a crucial function in the metabolism of fats and energy. The presence of bile acid in the bloodstream enables its direct interaction with the brain via the blood–brain barrier [107]. Additionally, it can modulate neuronal function via stimulating intestinal receptors, leading to the secretion of GLP-1 [108]. The interplay between the presence and elimination of bile acids is closely associated with the optimal functioning of the brain. The intestinal microbiota has the capacity to convert main bile acids into secondary bile acids via the use of many enzymatic pathways, including dehydrogenation, uncoupling, and degradation. The dysregulation of these neural circuits can result in several aberrant neurological processes, including neuroinflammation, impaired cognitive abilities, seizures, and demyelination. Secondary bile acids can be discovered in the brains of people suffering from AD, and the rise in the content is positively connected to cognitive impairment and brain imaging alterations [109]. In a clinical trial, it was observed that AD patients exhibited a significant decrease in the concentration of cholic acid, which is the main bile acid (BA), compared to non-AD patients. Additionally, AD patients showed elevated concentrations of deoxycholic acid, a secondary BA generated by bacteria activity, together with its glycine and taurine binding forms. The improved ratio of DCA to CA is indicative of a close association with cognitive decline, suggesting that the 7α-dehydroxylation pathway is implicated in this process [77].

### 3.2. The Integrity of Intestinal Barrier

The integrity of the gastrointestinal mucosa plays a crucial role in maintaining the general health of the body. The intestinal barrier is composed of three main components: intestinal microbes, intestinal mucosa, and tight junctions [110]. Tight junctions are a network of transmembrane protein strands that interact and connect adjacent cells, especially in immediate proximity to the adaptable surface of the epithelium [111]. This overview examines the impact of the intestinal mucosa and tight junctions on the physiological functioning of the human nervous system.

The intestinal mucosa, the innermost layer of the gastrointestinal tract, is composed of the epithelium, interstitial lamina propria with a rich vascular network, and muscularis mucosa. This complex structure is populated by specialized cells—absorptive epithelial cells, Paneth cells, goblet cells, and enteroendocrine cells—that together uphold the intestinal barrier’s integrity, demarcating the lamina propria from the luminal contents. Goblet cells secrete mucins, forming a mucus layer that acts as a gelatinous shield, limiting solute and water permeation and protecting epithelial cells from direct exposure to luminal entities. Intercellular junctions are crucial in maintaining these cells’ polarity and barrier impermeability.

Disruptions in the intestinal microbiota can alter this barrier, potentially resulting in endotoxemia, which is implicated in the etiology of chronic conditions such as obesity, type 2 diabetes mellitus, and inflammatory bowel disease. In the neurologic realm, a hypothesized link between Alzheimer’s disease and pathogens such as herpes simplex virus type 1, Chlamydophila pneumoniae, and Porphyromonas gingivalis suggests these microorganisms can traverse compromised barriers—intestinal and blood–brain alike—gaining access to the central nervous system and initiating neuroinflammatory responses that may contribute to neuronal damage.

Mind persistent inflammation can subsequently contribute to the development of peripheral nerve inflammation. The composition of the gut microflora consists of intricate colonies of microorganisms. There has been a hypothesis suggesting a possible link between AD and infectious agents such as herpes simplex virus type 1, Chlamydia pneumoniae, and Porphyromonas gingivalis, potentially influencing the development and progression of AD. Rotenone was employed as an agent to create the pathological model of PD in murine subjects in certain investigations. Following an extended period of administering the novel medicine FLZ, a notable decrease in both intestinal inflammation and damage to the intestinal barrier was observed in mice. Moreover, the treatment exhibited inhibitory effects on systemic inflammation. In addition, the administration of FLZ therapy has been shown to ameliorate microbiota imbalance and exert a neuroprotective effect via modulation of the TLR4 pathway [112]. The study showed that a one-month fasting period had a positive impact on the anxiety behavior of db/db mice over a three-month period. Additionally, the study observed a growth in the villus length of intestinal goblet cells and the thickness of the intestinal barrier muscle layer. Furthermore, the findings illustrated that IF may straightly act on intestinal fistula by reducing plasma lipopolysaccharide (LPS) levels [65]. Animal tests have also shown that in 4-month-old db/db mice, fasting every other day with a duration of 7 months led to an increase in intestinal mucin, goblet cells, and villous strips, while there is a decrease in plasma peptidoglycans. The aforementioned findings collectively indicate that IF has the potential to enhance the maintenance of the integrity of the intestinal barrier [67]. Simultaneously, SCFAs act as the primary energy substrate for intestinal epithelial cells, thereby facilitating the proliferation and differentiation of these cells, mitigating cell death, and preserving the integrity of the intestinal mucosal barrier. Numerous studies in both clinical and animal experimentation have consistently highlighted the capacity of various IF protocols to elevate concentrations of SCFAs inside the host organism. Additionally, these fasting regimens have been found to promote a greater biological abundance of the GIT, which serves as a significant source of SCFA production [113]. IF has been suggested as a potential strategy for enhancing the integrity of the intestinal barrier through its positive effects on gastrointestinal function, promotion of improved intestinal barrier function, reduction in the influx of detrimental substances into the intestine, and augmentation of intestinal immune cell activity.

### 3.3. Vagal Nerve Signal

The intrinsic neural network within the GIT, comprising the myenteric and submucosal plexus as well as Cajal interstitial cells, is primarily regulated by the sympathetic nervous system. This system exerts inhibitory effects on the secretion of gastrointestinal muscle and mucosa, and also modulates gastrointestinal blood flow via neuro-dependent vasoconstriction. In contrast, it can be argued that the parasympathetic nervous system exerts a more intricate and sophisticated control over the steady-state regulation of stomach, intestinal, and pancreas functions. Gut microbiota has the ability to interact via endocrine and immunological pathways, but its impact on the brain is most pronounced and expeditious when influencing the vagal nerve signal. The vagus nerve (VN) is a prominent constituent of the parasympathetic nervous system, encompassing approximately 80% of the afferent fibers and 20% of the penetrating fibers [114]. The metabolites of the microbiota could transmit information from the intestines to the CNS via afferent neurons. This process leads to the generation of adaptive or maladaptive responses following integration within the CNS [115]. Direct interaction between the gut microbiota and the afferent fibers of the VN was not established. The transmission of microbiota signals to these fibers occurs indirectly, primarily by the diffusion of microbial metabolites, chemicals, or other cells within the epithelium that are capable of transmitting signals. Within this group, certain substances such as SCFAs, gastrointestinal hormones, and endocrine peptides, including neuropeptide Y, peptide YY, pancreatic polypeptide, cholecystokinin, glucagon-like peptide, adrenocorticotropin releasing factor, oxytocin, and auxin releasing peptide, are thought to be involved in signaling transmission [116]. Several studies demonstrated that the prolonged administration of Lactobacillus rhamnosus JB-1 decreased the prevalence of anxiety and depression. Nevertheless, the vagotomy subordinates did not exhibit any alterations in behavior or physiological markers, so suggesting the significance of the VN as a crucial communication pathway within the brain–gut axis [117]. The administration of Lactobacillus reuteri demonstrated a specific enhancement in the social impairment observed in the mouse model of hereditary and idiopathic ASD. This effect is contingent upon the involvement of the VN. It has been seen that reuteri exerts its effects through a mechanism that relies on the VN. This mechanism has been found to restore synaptic plasticity in the ventral tegmental area of mice with ASD following social interaction [118]. However, it should be noted that this restorative effect is not observed in animals that lack oxytocin receptors. The current state of clinical research investigating the impact of IF on the intestinal VN, namely through modulation of gut flora, remains inconclusive. In this context, the author posits that IF potentialy influence the composition and diversity of microbiota, leading to alterations in VN signaling via the dissemination of microbial metabolites. Consequently, these changes might have an impact on the host’s neurological system and cognitive capabilities.

**Figure 3:**
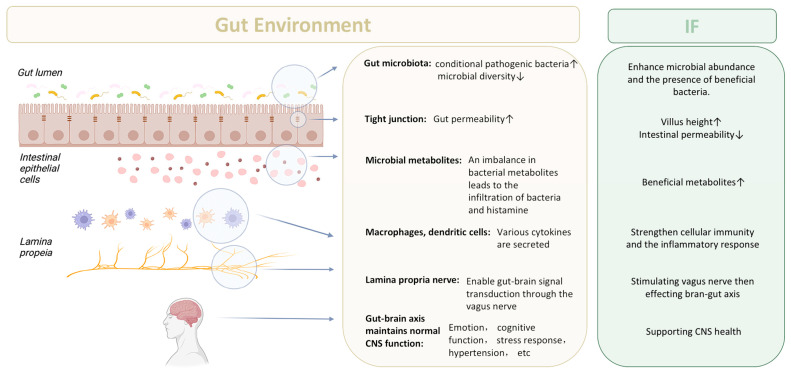
IF influences the intestinal environment and acts on the brain–gut axis. The gut environment is altered in a variety of ways by IF, including the kinds and quantities of intestinal bacteria, the intestinal barrier, and the gut nervous system. IF also regulates the gut environment through the gut–brain axis, which is a circular pathway that connects the gastrointestinal tract to the brain. The arrows in the figure correspond to increases or decreases in various factors related to the gut environment and the effects of IF. CNS—centre nervous system.

In Figure 3, we summarize the potential mechanism underlying the effect of IF on gut environment and brain–gut axis through gut microbiota and its metabolites.

### 3.4. Geographical Evaluation of IF and Gut Microbiota Studies across Diverse Populations

Evaluating the geographical regions where studies on IF and gut microbiota were conducted is indeed an insightful approach. Different regions can have varying dietary habits, environmental factors, and genetic backgrounds, all of which can significantly influence the gut microbiota [119]. This approach not only broadens the scope of the research but also emphasizes the importance of considering regional factors in interpreting and applying study findings, especially in the context of globally diverse dietary practices and environmental influences.

To evaluate the geographical areas of gut microbiota studies, research by location, ensure the inclusion of diverse populations, and analyze regional diets and environmental factors should be gathered and categorized. Then, examine lifestyle and health factors across these regions, apply statistical analysis to identify patterns or correlations, and finally, synthesize and report findings, highlighting any research gaps and the potential influence of geography on the gut microbiota. The changes of gut microbiota in different regions and ethnic groups after IF are worthy of further investigation.

## 4. The Potential Role of Gut Microbiota in IF-Mediated Neuroprotection

We investigated the role of the gut microbiota in elucidating the protective effects and underlying mechanisms of IF in the context of various situations associated with neurological diseases.

### 4.1. IF and Neurodegenerative Diseases

IF has been investigated in a variety of neurodegenerative disease models. In Table 4, we summarize the outcomes of IF on various neurodegenerative conditions, detailing the model systems and the durations of the interventions.

#### 4.1.1. Alzheimer’s Disease

Recent research conducted on rodents has indicated that changes in the gut microbiome were potentially associated with the accumulation of amyloid deposits, which are associated with AD [120,121]. Yet, the specific microbial profiles associated with AD in humans remain unclear. In an effort to delineate these profiles, researchers analyzed fecal samples from individuals with and without AD-induced dementia. The studies revealed a reduced microbial diversity in the AD-diagnosed group, characterized by distinct compositional differences from age- and sex-matched controls. Notably, researchers identified shifts in bacterial abundance at various taxonomic levels, with reductions in Firmicutes, increases in Bacteroidetes, and a decline in Bifidobacterium. These microbial changes correlated with AD biomarkers in cerebrospinal fluid (CSF), bolstering the evidence that gut microbiota alterations are linked to AD and other disorders. The findings also suggest that modulating gut bacteria could serve as a therapeutic strategy [122].

Alterations in gut microbiota may trigger pro-inflammatory cytokine release and increase intestinal barrier permeability, potentially leading to insulin resistance, a known AD correlate [123]. Moreover, the gut microbiome is known to release immunogenic combinations of amyloids, lipopolysaccharides (LPSs), and other microbial exudates into its immediate surroundings [124,125,126,127,128,129]. Bacterial amyloids might activate signaling pathways known to play a role in neurodegeneration and AD pathogenesis, while the gut microbiome might enhance inflammatory responses to cerebral accumulation of amyloid-beta (Aβ) [130]. Previous research has proposed that the composition of the gut microbiota can be influenced by diet and certain nutrients [131]. Furthermore, it has been claimed thatthese factors may have an impact on the formation or aggregation of amyloid proteins [130,132,133,134,135]. This suggests that manipulating the gut microbiome via targeted nutritional interventions and utilizing prebiotics and probiotics could potentially prevent or alleviate symptoms of AD [136].

**Table 4:** Impact of IF on various neurodegenerative diseases.

Diseases	Outcomes of IF	Model System	Duration of Intervention	Key Findings
Alzheimer’s Disease	Exacerbated AD-like neurodegenerative changes	5XFAD mice	4 months	Every other fasting regimen increased inflammation and altered glutamatergic signaling, without affecting Aβ load [137].
	Enhanced Aβ clearance through autophagy	In vitro (neuronal toxicity)	Not specified	Caloric restriction and prolonged IF increased markers of autophagic activity and decreased markers of apoptosis [138].
	Improvement in neuronal differentiation and memory	3xTg-AD mice	3 months	IF activated GSK-3β, leading to enhanced neuronal differentiation in the hippocampus and improved memory [139].
	Neuroprotective effects; increased BDNF and NT3	Type 2 diabetic rats	3 months	IF increased levels of BDNF, NT3, serotonin, dopamine, and glutamic acid, showing potential protective effects [140].
	Improved cognitive function and Aβ clearance	APP/PS1 double-transgenic mice	5 months	IF restored AQP4 polarity, possibly through β-hydroxybutyrate, leading to reduced Aβ pathology [141].
	Ameliorated cognitive deficits and reduced Aβ and tau pathologies	3xTgAD mice	7 or 14 months	Both CR and IF improved cognitive function, with CR showing reduced Aβ and tau levels [142].
	Improved memory function and alleviated osteoarthritic symptoms	Ovariectomised rats induced with AD and OA	6 weeks	IF with a high-protein diet showed neuroprotective effects, potentially through the gut–microbiota–metabolites–brain axis [143].
	Improved cognitive function and metabolic disturbances	Ovariectomized rats infused with β-amyloid	4 weeks	IF protected against memory loss and metabolic disturbances in estrogen-deficient rats [144].
Parkinson’s Disease	Neuroprotective; reduced dopaminergic neuronal loss and astroglial activation	MPTP mouse model	2 weeks	Alternate-day fasting increased neurotrophic factors, suppressed motor impairments, and mitigated MPTP-induced dopaminergic neuronal loss [145].
	Exacerbated neuronal death and increased excitatory amino acids	C57BL/6J mice treated with rotenone	28 days	IF in combination with neurotoxin exposure led to increased neuronal death, excitatory amino acids, and inflammatory lipids [146].
Huntington’s Disease	Enhanced mHTT clearance and promoted autophagy	YAC128 mice expressing cleavable mHTT	Not specified	Scheduled feeding paradigm reduced mHTT levels; fasting-induced autophagy remained functional despite impaired basal autophagy due to cleavable mHTT [147].
Multiple Sclerosis	Investigate impact on MS during Ramadan	80 adult MS patients (40 fasting, 40 non-fasting) in Isfahan, Iran	Ramadan period + 6 months follow-up	No significant changes in disability or clinical relapses [148].
	Determine feasibility of Time Restricted Eating (TRE)	12 participants with RRMS	8 weeks, 16 h fasting daily	TRE feasible and acceptable; exploratory results suggest further study warranted [149].
	Assess intermittent caloric restriction on EAE (MS model)	Mice with EAE	4th week post-immunization, two cycles of 3 days FMD + 4 days normal feeding	Decreased EAE severity, immune cell infiltration, and CNS demyelination; enhanced CNS recover [150].
	Explore effects of IF on MS and EAE, focusing on gut microbiota	EAE mice and pilot clinical trial in MS patients	/	IF improved clinical course and pathology in EAE, altered gut flora and T cell profiles; similar effects in pilot clinical trial [47].

Aβ—amyloid β-protein; AD—Alzheimer’s disease; CR—calorie restriction; EAE—experimental autoimmune encephalomyelitis; IF—intermittent fasting; MS—multiple scerosis; OA—osteoarthritis.

The primary risk factor for AD is the process of aging, and implementing dietary energy restriction has been demonstrated to slow down the aging processes in the brain [142]. While IF’s efficacy as an intervention against age-related metabolic dysfunction remains debated [151], studies have assessed its effects on metabolic and cognitive decline in estrogen-deficient rats, exploring the underlying mechanisms. The study included four groups: AD with unrestricted feeding, AD with IF, non-AD with unrestricted feeding, and non-AD with IF. Rats in the IF groups underwent restricted feeding to a 3 h window daily. All rats, totaling ten per group, were fed a high-fat diet over four weeks. The findings revealed that IF moderated AD-associated increases in tail skin temperature and abdominal fat mass, improved energy balance via food intake without altering energy expenditure, and favored fat oxidation over glucose. In the context of AD, IF also alleviated memory loss and reduced serum glucose levels post-glucose challenge by enhancing insulin secretion. Moreover, IF lowered cortisol levels and improved dyslipidemia and liver health compared to unrestricted feeding. However, IF also heightened bone mineral density loss and insulin resistance during fasting [144].

#### 4.1.2. Parkinson’s Disease (PD)

Parkinson’s disease, a neurodegenerative condition, affects various brain regions and is marked by α-synuclein accumulation in the CNS [152,153]. It can disrupt components of the brain–gut axis, including the autonomic and enteric nervous systems [154,155]. In addition, it is also recognized that the dysregulation of the brain–gut–microbiota axis in PD may lead to gastrointestinal (GI) dysfunction, a prevalent condition affecting over 80% of individuals with PD [156]. There’s growing evidence that GI dysfunction may be integral to PD pathogenesis, possibly initiating in the gut and progressing to the brain [157]. Studies have shown that antibiotics can exacerbate aSyn pathology in mice, and gut microbiota manipulation can accelerate this process. These insights underscore the regulatory role of gut–brain communication in PD development. Transplanting microbiota from healthy individuals to aSyn-overexpressing mice resulted in less damage compared to microbiota from PD patients, highlighting the role of gut bacteria in PD and suggesting that human microbiome alterations are risk factors for the disease [158].

Further, there is a potential link between changes in the human microbiome and PD risk. Investigations into PD patients, animal models, and genetic mutations causing familial PD indicate impaired neuronal bioenergetics, particularly in brainstem and midbrain monoaminergic neurons [159,160]. The presence of peripheral insulin resistance and diabetes during middle age has been found to potentially elevate the likelihood of PD [161,162,163]. However, it has been suggested that making changes to one’s diet and lifestyle that enhance insulin sensitivity, such as engaging in regular exercise [164,165,166] and conducting intermittent energy restriction [167,168,169,170], may potentially mitigate neurodegenerative mechanisms and result in enhanced functional outcomes in animal models. The utilization of insulin-sensitizing GLP-1 analogs has demonstrated advantageous effects in animal models of PD. Furthermore, the preliminary findings from an initial clinical trial including PD patients have shown promise. In addition to their beneficial effects on peripheral and brain energy metabolism, exercise, intermittent energy restriction, and GLP-1 analogs have been observed to increase neurotrophic signaling, DNA repair, proteostasis, and mitochondrial biogenesis as part of the neural adaptive stress response [171].

#### 4.1.3. Huntington Disease (HD)

Huntington’s disease is characterized by a unique phenotype that encompasses chorea and dystonia, impaired coordination, cognitive deterioration, and behavioral challenges. This condition follows an autosomal-dominant inheritance pattern and exhibits increasing manifestations over time [172]. A study reported a 41-year-old male patient who diagnosed HD and received a 48-week time-restricted ketogenic diet. The individual showed improvement in motor symptoms, performance of daily tasks, cUHDRS score, predominant HD-associated behavioral issues, and mood-related quality of life, but no improvement in cognition [173]. Metabolic approaches, such as fasting and ketogenic diets, were found could ameliorate the clinical manifestations of HD by enhancing brain and muscle metabolism and improving mitochondrial function [174]. A preclinical research supported the therapeutic potential of IF techniques based on circadian rhythms. After a period of three months of treatment, the BACHD micemodel, which is characterized by the manifestation of many fundamental symptoms of HD, demonstrated enhancements in both locomotor activity and sleep behavioral cycles subsequent to treatment with TRF. In addition, there was an observed increase in heart rate variability, indicating a potential amelioration of autonomic nervous system dysfunction [175]. Another kind of HD mouse model, the heterozygous Q175, which shows many HD core symptoms was also used to verify the association between IF and HD. Q175 mice treated with TRF for three months showed improvement in locomotor activity rhythm, sleep-wake time and heart rate variability. By gene expression analysis, several HD-related markers were found to return to wild-type levels in the striatum of treated mice [176]. Currently, there is no direct evidence linking IF to the progression of PD via gut microbiota. However, given that the gut expresses mutant HTT and HD pathology is present in the ENS of HD patients and mouse models [177,178], it is plausible that IF influences HD progression by modulating gut flora, thereby affecting the ENS or directly engaging in host metabolism.

#### 4.1.4. Multiple Sclerosis (MS)

Multiple sclerosis, a chronic autoimmune disease, is characterized by CNS demyelination and affects 2.5 million people worldwide, with profound personal and societal consequences [179]. It may present as relapsing-remitting MS or a progressive form with gradual neurological decline. Central immune response dysfunction is a key mechanism in MS, where gut microbiota imbalance can provoke a localized immune response. Activated immune cells may cross the blood–brain barrier, leading to abnormal central immune reactions. Intestinal microbiota could influence MS onset and progression by disturbing CNS immune responses, metabolic processes, intestinal barrier integrity, and blood-brain barrier.

The experimental autoimmune encephalomyelitis (EAE) model is pivotal in advancing MS therapies [180]. CD4+ T cells play a crucial role in experimental EAE and are believed to contribute to the pathogenesis of MS. CD4+ T helper (Th) cells exhibit unique cytokine profiles and express master transcription factors, which are employed to delineate several T cell subsets [181]. Multiple lines of evidence suggest that CD4+ T cells that produce IL-17 (known as Th17 cells), interferon (IFN)-γ (known as Th1 cells), and granulocyte-macrophage colony-stimulating factor (GM-CSF) have a pathogenic role in MS and experimental EAE. On the other hand, regulatory T cells possess immunomodulatory and protective properties. Numerous studies underscore the significance of the intricate interaction among diet, metabolic status, and immune-inflammatory responses in the context of MS [182]. Obesity’s link to autoimmunity might stem from a persistent low-grade inflammatory state with altered adipokine production [183]. The gut microbiota could induce pro-inflammatory or anti-inflammatory responses by modulating T cell differentiation and immune responses within the GIT. Studies in the EAE model show that this phenomenon may have significant systemic implications in either exacerbating or providing protection against autoimmune disorders [184,185,186,187,188]. Recently, it was documented alterations in the gut microbiota of patients with relapsing-remitting MS as compared to individuals without the condition [189]. Further, CR exhibits significant anti-inflammatory effect. Previous research indicated that chronic CR effectively prevented experimental EAE development [190]. However, chronic CR may not be suitable for most people. IF induces similar physiological responses and could be a more feasible dietary intervention. A short pilot study demonstrated the feasibility and safety of an IF relapse intervention, leading to short-term metabolic and gut microbiome changes akin to those in animal models [47].

### 4.2. IF and Acute Central Nervous System Injury

#### 4.2.1. Ischemic Stroke

Ischemic stroke, a prevalent condition with limited treatment options, involves inflammation in its pathogenesis [191,192]. The presence of commensal gut microbiota has was found to impact the immune system and potentially influenced disease processes in the brain [192,193]. Studies showed that antibiotics-induced intestinal microbiota alterations reduced ischemic brain injury in mice, with benefits via fecal transplantation. Dysbiosis in the small intestine disrupted immune balance, upregulated regulatory T cells and downregulated IL-17–positive γδ T cells and consequently impeded effector T cell migration to the leptomeninges post-stroke [194]. The neuroprotective effects of dysbiosis require the presence of IL-10 and IL-17 [195,196]. The study confirmed the role of gut–brain axis in ischemic injury.

Several studies uncovered a new inflammatory mechanism that played a role in tissue damage during cerebral ischemia. This process involves the activation of inflammasomes, which are multi-protein complexes [197]. IF has been shown to have the potential to reduce the concentrations of pro-inflammatory cytokines both in the peripheral tissues and the brain. In this study, we examined the effects of IF, which involved a daily period of 16 h of food deprivation, over a four-month duration, on the activity of NLRP1 and NLRP3 inflammasomes following cerebral ischemia. The experimental procedure involved the induction of ischemic stroke in C578L/6J mice through the closure of the middle cerebral artery, followed by subsequent reperfusion. The administration of IF decreased the activation of the NF-kappa B and MAPK signaling pathways, as well as the expression levels of the NLRPI and NLRP3 inflammasome proteins, as well as IL-1 beta and IL-18, in ischemic brain tissue. After an ischemic stroke, the outcomes of this study indicate that IF may reduce the inflammatory response and tissue injury. This effect is achieved by suppressing the activity of NLRP1 and NLRP3 inflammasomes [198].

#### 4.2.2. Epilepsy

A prospective observational training was managed to examine the impact of fasting during Ramadan in 2019 on Muslim patients with active epilepsy. This study focused on individuals who intended to fast and involved an average fasting duration of 16 h per day and monitored the frequency of seizures for each type of seizure over a period of three months. The result from this study indicated that Ramadan fasting might be beneficial in active focal, myoclonic, and absence seizures, as well as a post-fasting effect [199]. Furthermore, it showed that IF probably facilitated axonal regeneration following the compression of the sciatic nerve in mice by regulating of the intestinal microbiome and its corresponding metabolite indole-3-propionic acid. The validation of this phenomenon has been conducted using the mouse sciatic nerve injury model, and additional investigation in the CNS is required to expand our comprehension.

### 4.3. Perioperative Neurocognitive Dysfunction (PND)

As the accelerating global aging, a growing number of senior individuals are having anesthetic and surgical procedures [200]. As a result, there is a growing focus on perioperative neurocognitive dysfunction [201]. There was a robust correlation between gut microbiota and PND, which is related to the gut–brain axis in our and other’s previous studies [202,203,204,205]. Perioperative fasting is a common way of perioperative nutrition management [206]. A recent study found preoperative fasting protected against intestinal ischemia/reperfusion injury by modulating gut microbiota [207]. Our recent study also showed that preoperative nutritional status could predict postoperative renal injury [208]. However, until now, there is no sufficient evidence on whether IF influences the outcome of PND. It is worth further exploring whether pre-operative short-term IF, long-term IF, or postoperative IF impacts cognitive function via intestinal flora.

## 5. Discussion

This review has synthesized insights into the potential role of IF in neurological disorders, highlighting the pivotal role of gut microbiota. The integrity of intestinal mucosa and tight junctions is critical for the physiological functioning of the nervous system. Epithelial cells, goblet cells, and intestinal endocrine cells within the mucosal layers are integral in maintaining the structural integrity of the intestinal barrier. Disturbances in the gut flora lead to increased intestinal epithelial permeability, resulting in endotoxemia, which is closely linked to the development of chronic diseases such as obesity, type 2 diabetes, and inflammatory bowel disease. Moreover, there is a hypothesized connection between gut flora and the nervous system, suggesting that certain pathogens may cross the compromised intestinal barrier and induce neuroinflammatory responses and nerve damage.

IF emerges as a potential strategy to bolster intestinal barrier integrity, demonstrating benefits such as improved gastrointestinal function, enhanced intestinal barrier function, reduced entry of harmful substances, and bolstered activity of intestinal immune cells. IF may also elevate concentrations of short-chain fatty acids (SCFAs), which are vital for the proliferation and differentiation of intestinal epithelial cells, reducing cell apoptosis, and maintaining the integrity of the intestinal mucosal barrier.

Nevertheless, the exploration of IF’s influence on neurological diseases through gut microbiota necessitates further research in several areas:

Human clinical trials: The evidence base would benefit from more robust human clinical trials to substantiate the effects of IF on neurological disorders.

Long-term studies: There is a scarcity of long-term investigations to understand the sustained effects and potential side effects of IF on neurological health.

Diverse populations: Research predominantly focuses on healthy or obese individuals, with a dearth of studies on specific populations such as the elderly, children, or those with neurological diseases, among whom the effects of IF may differ.

Mechanistic explanations: While the impact of IF on gut flora is acknowledged, in-depth explanations of the mechanisms by which IF influences neurological diseases are lacking.

Standardized IF protocols: With a variety of IF protocols in existence, comparative studies are necessary to discern the most effective methods for neurological disorders.

Despite the growing interest in IF’s potential role in neurological diseases, these gaps underscore the need for further research to elucidate its effects and mechanisms. The study outlines the following key future research directions:

A comprehensive examination of the relationship between gut microbiota and neurodegenerative diseases, including Alzheimer’s.

Investigation into the microbiota-mediated neurotransmission mechanisms, especially the effects of metabolites like SCFAs on the nervous system.

Analysis of IF’s preventive and therapeutic potential for neurological disorders, probing into its mechanisms and clinical applications.

Strategies for regulating intestinal flora, using probiotics, prebiotics, flora transplantation, and assessing their impact on the nervous system.

Further understanding of the gut–brain axis, exploring the role of intestinal flora, the mucosal barrier, neurotransmission, and other factors in gut–brain communication.

## 6. Conclusions

IF has prompted changes in the intestinal microflora composition and metabolite production, influencing the integrity of the intestinal barrier and peripheral nervous system function. These alterations could affect physiological and psychopathological processes, even in individuals without preexisting health issues. The findings provide a basis for developing therapeutic interventions and call for normative clinical studies to examine the effects of different dietary regimens on gut microbiota. Advancing our understanding of intestinal flora through various methodologies, including microbiota detection, could inform the translation of IF protocols into clinical practice with significant potential.

## Figures and Tables

**Table 1:** Studies examining the effect of fasting on intestinal flora in human.

Author, Year	Number of People	Fasting Plan	Sample Type	Microbiological Analysis Method	Main Findings
Duygu Saglam, 2023	12 adults	TRF for 29 days	Feces	16s rRNA	Several gut bacteria including Firmicutes ↓ *Escherichia* and *Shigella* ↑ [34]
Xiangwei Hu, 2023	72 adults	PF(5:2 diet) lasting for three weeks	Feces	16s rRNA	*Parabacteroides distasonis* and *Bacteroides thetaiotaomicron* ↑ [35]
Alex E Mohr, 2022	20 obese adults	Two groups, one fasted one day a week with six days of low calorie, the other fasted two days with five days of low calorie	Feces	16s rRNA	*Ruminal coccidiaceae* ↑ [36]
Muhammad Nadeem Khan, 2022	14 women and 31 men	TRF for 26 days	Feces	16s rRNA	Anti-inflammatory *bacteria Lactobacillus* and *Bifidobacterium* were favorably ↑, while pathogenic bacteria were ↓ [37]
Junhong, Su, 2021	34 overweight people	TRF for a month	Feces	16s rRNA	Microbial diversity ↑ Especially *Spriochetes* from *Clostridia* ↑ [38]
Maggie A, 2021	34 overweight people	ADF for three months	Feces	16s rDNA	Microbial species and abundance ↑ [39]
Guo Yi, 2021	39 patients with MS	2-day modified IF for 8 weeks	Feces	16s rRNA	The community structure of intestinal flora was not significantly affected *Acidobacter* ↓ [40]
Mohanmmadzaheh A, 2021	30 healthy examiners	Fasting in Ramadan(TRF) for a month	Fasting blood sample	16s r RNA, qPCR	Bacteria that can degrade dietary ↓ [41]
Maifeld A, 2021	63 patients with METs	12 Weeks, 300–350 kcal five days a week and modified DASH diet for the rest days	Feces	16s rRNA gene	Changes in the diversity of the gut microbiome [42]
Hassane Z, 2020	30 obese males	Fasting in Ramadan(TRF) for a month	Blood sample	qPCR	Serum leptin level ↑, GLP- ↓, PYY ↓, CCK ↓ [43]
Gabel K, 2020	14 obese adults	TRF for 12 weeks	Feces	16s rRNA gene	The systemic diversity of intestinal microbiota remains unchanged [44]
Mesnage R, 2019	15 healthy males	Buichinger fasting plan for 10 days with enema every two days	Feces	16s rRNA gene amplification	Bacteria that can degrade dietary polysaccharides (such as *Trichospiridae* and *Ruminococcaceae*) ↓ [45]
Ozkul C, 2019	9 adults	Fating in Ramadan(TRF) for 29 days,	Feces	16s rRNA gene, qPCR analysis	*Coliphage* and *Bacteroides fragilis* ↑ [46]
Cignarella F, 2018	16 patients with MS	ADF for 15 days, daily intake less than 500 kcal	Feces	16s rRNA gene	*Faecali bacterium* ↑ [47]

16s rRNA—16S ribosomal RNA gene sequencing; ADF—alternate day fasting; IF—intermittent fasting; MS—metabolic syndrome; PF—periodic fasting; TRF—time-restricted feeding; ↑—increase; ↓—decrease.

**Table 2:** Fasting on intestinal flora in animal studies.

Author, Year	Research Model	Fasting Plan	Sample Type	Microbiological Analysis Method	Main Findings
Sainan Wang, 2023	5-week-old C57 male mice	ADF	Feces	16s rRNA gene	Firmicutes to Bacteroidetes ratio ↓ [48]
Junyu Wang,2023	2-month-old Sprague-Dawley rat	ADF	Feces	16s rRNA gene	Variation in gut microbiota’s abundance and diversity with alternate-day fasting in injured rats [49].
Hikmet Taner Teker, 2023	12-month-old Wistar male rat	TRF for 35 days	Cecum regions and their contents	16s rRNA gene	The Firmicutes and Bacteroidetes ratio ↓, Proteobacteria ↓ [50]
Jiafeng Xia, 2023	6-week-old C57 male mice	Four groups were: unrestricted Western diet, time-restricted Western diet, unrestricted chow diet, and time-restricted chow diet	Feces	16s rRNA gene	The TRF scheme restored the rhythmicity of genera such as Lactobacillus, Myxococcus and Acetobacter [51].
Ruxue Ma, 2023	6–8-week-old Balb/c female mice	Three groups for four weeks: TRF, ADF, normal feeding	Feces	16s rRNA gene	The expression of ZO- is higher in the ileum of two IF groups of mice. / hour fasting led to a reconfiguration of the gut microbiota. Alistipes and Rikenellaceae ↑ [52]
Junhong Su, 2022	6-week-old Balb/c male mice	TRF for 30 days	Feces	16s rRNA	Lachnospiroceae and Ruminococcaceae ↓ [53]
Ruiyuan Pan, 2022	3-month-old 5xFAD mice	ADF until the mice grow to 5.5–6 months old	Feces	16s rRNA gene	Firmicutes phylum ↑ Bacteroidetes ↓ Lactobacillus family ↑ [54]
Yang Hong, 2023	6-week-old C57 male mice	ADF	Feces	16s rRNA gene	Lactobacilli and Verrucomicroniaceae ↑ [55]
Jingjing Wu, 2022	6-week-old C57 male mice	Short-term group: IF for two weeks; Long-term group: IF for 20 weeks	Feces	Non-targeted sequencing	Short-term IF: Colitogenic Bacteroides, Dopakuru and Akmansia ↑, Clostridium ruminant ↓. long term IF: Akmansia↓ and Lactobacillus ↑ [56]
Hernandez Abii R, 2022	8-month-old mice	TRF(Fasting 21 h a day) for 14 months	Feces	16s rRNA gene amplification	Genus Leptomyces, Enteromonas and Eubacterium ↑ [57]
Andras G, 2022	C57 male mice	IF group: fasting every other day for 28 days; FMD group: fasting four days a week for three weeks	Cecal contents	16s rRNA gene	SCFAs level ↓ [58]
Shanshan Xie, 2022	12-week-old c57 male mice		Feces	16s rRNA gene	The level of distant Bacteroides ↓ [59]
Huanan Shi, 2021	5-week-old hypertensive rats	ADF for 10 weeks	Feces	16s rRNA gene	Lactobacillus and Bifidobacterium ↑ [60]
Ziyi Zhang, 2021	8-week-old c57 male mice	PF(5:2 IF regimen)	Feces	16s rRNA gene	Lactobacillus ↓ [61]
Jingliang Liu, 2021	6-week-old c57 male mice	1. Control group; 2. Intermittent group; 3. Melatonin group; 4. Fasting with melatonin group	Feces	16s rRNA gene amplification	Short-term IF: colitis cells, Bacteroides, Dopakuru and Akmansia ↑ , and Clostridium ruminant ↓. Long term IF:Lactobacillus ↑, Akmansia ↓ [62]
Ya Deng, 2020	3-week-old c57 male mice	ADF for a month	Feces	16s rRNA gene amplification	The proportion of thick-wall bacteria and Bacteroides ↓ The relative abundance of intestinal flora [63]
Yuqian Ye, 2020	8-week-old Kunming male mice	Either a normal diet ad libitum, a high-fat diet ad libitum, or a high-fat diet restricted to TRF; for 8 weeks	Feces	16s rRNA gene	Bacteroidetes ↑ Firmicutes ↓ [64]
Zhigang Liu, 2020	3-month-old db/db mice	NA	Feces	16s rRNA gene	The integrity of intestinal barrier ↑ The level of SCFAs ↑ Intestinal microorganisms, and Lactobacillus ↑ Plasma LPS ↓ [65]
Linghao Li, 2020	C57 male mice	NA	Feces	16s rRNA	Akkermansia ↑ Alisma ↓ [66]
Cignarella F, 2018	7-week-old c57 BL/6 female mice	Fasting every other day for four weeks	Feces	16s rRNA gene	Lactobacilli, Bacteroides and Prevostiae ↑ [47]
Eleni B, 2018	4-month-old db/db mice	Fasting every other day for seven months	Feces	16s rRNA gene	The level of Bacteroides ↑, while Verruciformes ↓; The number of mucin, goblet cells, and villus length of intestinal mucosa in mice ↑, and plasma peptidoglycan ↓ [67]

16s rRNA—16S ribosomal RNA gene sequencing; ADF—alternate day fasting; IF—intermittent fasting; NA—no application; PF—periodic fasting; TRF—time-restricted feeding; ↑—increase; ↓—decrease.

**Table 3:** Effects of microbial metabolites on the nervous system.

Metabolites	Related Bacterium	Main Findings
SCFAs: acetic acid, propionic acid, butyric acid, isobutyric acid, etc.	*Clostridium* group of *Chlamydomonas*, including *Eubacterium*, *Rosbergia*, *Fecal*, etc.	1. Butyric acid passed GPR109A/PPAR-γ/TLR4-NF-κ B signal pathway inhibits microglia-mediated neuroinflammation and enhances memory and cognitive performance in a correlated manner [71].
		2. Supplementing SCFAs alleviated the manifestation of anxious and depression behaviors in mice [72]
		3. Chronic cerebral hypoperfusion resulted in the decrease in fecal acetic acid and propionic acid and the decrease in hippocampal acetic acid. After administration of FMT and SCFA, the above decrease was reversed by changing the structure and composition of fecal microbial community; FMT and SCFAs may alleviate the neuronal damage induced by chronic cerebral ischemia [73].
		4. After young adult mice were transplanted into the microbiota of old mice, the expression of synaptic plasticity and neurotransmission proteins in the hippocampus decreased, and the microbiota producing SCFAs (*Trichospiridae*, *Faecaceae* and *Ruminococcaceae*) decreased [71]
		5. In VPA-induced autism model rats, the abundance of uric acid bacilli is high and the level of butyric acid is low. *Lactobacillus suis* CCFM 1076 helps to decrease the prevalence of uric acid bacteria and enhance the concentration of butyric acid [74]
Choline metabolites	*Faecalibacterum prausznitzii*	1. TMAO is synthesized via the metabolic process of choline by intestinal microbes. The substance has the ability to traverse the blood-brain barrier and exert its effects on the central nervous system. The heightened level of focus will amplify the likelihood of experiencing unfavorable cardiovascular events [75].
		2. Individuals who have elevated choline levels demonstrate a reduced susceptibility to cognitive impairment subsequent to an ischemic stroke [76].
Cholic acid: cholate, porcine cholate, deoxycholate, etc.	*Faecalibacterium praussznitzii*, *Lactobacillus, Bifidobacterium*, etc.	1. Individuals diagnosed with AD have reduced blood concentrations of CA, which is a primary BA. Conversely, these individuals show elevated levels of DCA, a secondary bile acid that is generated by bacteria [77].
		2. There was a significant correlation seen between decreased concentrations of CDCA, CA, and UDCA, and the presence of PD-MCI [78].
Amino acid derivatives	*Faecalibacterium praussznitzii*, *Lactobacillus, Bifidobacterium*, etc.	1. Research investigations indicate that the suppression of intestinal ecological disturbances and the subsequent buildup of phenylalanine and isoleucine may effectively regulate neuroinflammation and alleviate cognitive impairment [79].
		2. Obese diet mice showed cognitive dysfunction, accompanied by intestinal ecological disorder and Trp metabolic disorder [80].
Vitamins: Vitamin K, Vitamin B12, thiamine, folic acid, etc.	NA	1. There exists a positive correlation between elevated consumption of vitamin K via one’s diet and enhanced cognitive performance [81].
		2. Elevated levels of plasma homocysteine have been illustrated to be positively correlated with an increased susceptibility to cognitive impairment and dementia [82].

AD—Alzheimer’s disease; BA—bile acid; CA—cholic acid; CDCA—deoxycholic acid; DCA—deoxycholic acid; NA—no application; PD-MCI—Parkinson’s disease with mild cognitive impairment; TMAO—trimethylamine oxide; UDCA—ursodeoxycholic acid; Trp—tryptophan.

## Abbreviations

4EPS	4-ethyphenyl sulfate
AD	Alzheimer’s disease
ADF	alternate day diet
ANS	autonomic nervous system
Aβ	amyloid-beta
CNS	central nervous system
CR	calorie restriction
CSF	cerebrospinal fluid
EAE	experimental autoimmune encephalomyelitis
ENS	intestinal nervous system
FMD	simulated fasting diet
GI	gastrointestinal
GIT	gastrointestinal tract
GLP-1	glucagon-like peptide-1
HD	Huntington’s disease
IER	immune effector response
IF	intermittent fasting
IL	interleukin
KD	ketogenic diet
LPSs	lipopolysaccharides
MS	multiple sclerosis
PD	Parkinson’s disease
PF	periodic fasting
PND	perioperative neurocognitive dysfunction
SCFAs	short-chain fatty acids
SHBG	sex hormone binding globulin
TRF	time restricted diet
VN	vagus nerve
cICCs	Cajal interstitial cell
αSyn	α-synuclein

## References

[1] Przybyłowicz K.E., Danielewicz A. (2022). Eating Habits and Disease Risk Factors. Nutrients.

[2] Mohan V., Unnikrishnan R., Shobana S., Malavika M., Anjana R., Sudha V. (2018). Are excess carbohydrates the main link to diabetes & its complications in Asians?. Indian J. Med. Res..

[3] Flanagan E., Lamport D., Brennan L., Burnet P., Calabrese V., Cunnane S.C., de Wilde M.C., Dye L., Farrimond J.A., Lombardo N.E. (2020). Nutrition and the ageing brain: Moving towards clinical applications. Ageing Res. Rev..

[4] Wilkinson M.J., Manoogian E.N.C., Zadourian A., Lo H., Fakhouri S., Shoghi A., Wang X., Fleischer J.G., Navlakha S., Panda S. (2020). Ten-Hour Time-Restricted Eating Reduces Weight, Blood Pressure, and Atherogenic Lipids in Patients with Metabolic Syndrome. Cell Metab..

[5] Fanti M., Mishra A., Longo V.D., Brandhorst S. (2021). Time-Restricted Eating, Intermittent Fasting, and Fasting-Mimicking Diets in Weight Loss. Curr. Obes. Rep..

[6] Stockman M.-C., Thomas D., Burke J., Apovian C.M. (2018). Intermittent Fasting: Is the Wait Worth the Weight?. Curr. Obes. Rep..

[7] Gudden J., Vasquez A.A., Bloemendaal M. (2021). The Effects of Intermittent Fasting on Brain and Cognitive Function. Nutrients.

[8] Cryan J.F., O’Riordan K.J., Sandhu K., Peterson V., Dinan T.G. (2020). The gut microbiome in neurological disorders. Lancet Neurol..

[9] Whittaker D.S., Akhmetova L., Carlin D., Romero H., Welsh D.K., Colwell C.S., Desplats P. (2023). Circadian modulation by time-restricted feeding rescues brain pathology and improves memory in mouse models of Alzheimer’s disease. Cell Metab..

[10] de Vos W.M., Tilg H., Van Hul M., Cani P.D. (2022). Gut microbiome and health: Mechanistic insights. Gut.

[11] Furness J.B. (2012). The enteric nervous system and neurogastroenterology. Nat. Rev. Gastroenterol. Hepatol..

[12] Hofer U. (2022). Gut–brain axis in ageing. Nat. Rev. Microbiol..

[13] Welton S., Minty R., O’Driscoll T., Willms H., Poirier D., Madden S., Kelly L. (2020). Intermittent fasting and weight loss Systematic review. Can. Fam. Physician.

[14] Varady K.A., Cienfuegos S., Ezpeleta M., Gabel K. (2022). Clinical application of intermittent fasting for weight loss: Progress and future directions. Nat. Rev. Endocrinol..

[15] De Cabo R., Mattson M.P. (2019). Effects of Intermittent Fasting on Health, Aging, and Disease. N. Engl. J. Med..

[16] Mattson M.P., Moehl K., Ghena N., Schmaedick M., Cheng A. (2018). Intermittent metabolic switching, neuroplasticity and brain health. Nat. Rev. Neurosci..

[17] Mattson M.P., Longo V.D., Harvie M. (2017). Impact of intermittent fasting on health and disease processes. Ageing Res. Rev..

[18] Karam G., Agarwal A., Sadeghirad B., Jalink M., Hitchcock C.L., Ge L., Kiflen R., Ahmed W., Zea A.M., Milenkovic J. (2023). Comparison of seven popular structured dietary programmes and risk of mortality and major cardiovascular events in patients at increased cardiovascular risk: Systematic review and network meta-analysis. BMJ.

[19] Pedersen A.L.W., Lindekilde C.R., Andersen K., Hjorth P., Gildberg F.A. (2021). Health behaviours of forensic mental health service users, in relation to smoking, alcohol consumption, dietary behaviours and physical activity—A mixed methods systematic review. J. Psychiatr. Ment. Health Nurs..

[20] Gill S.K., Rossi M., Bajka B., Whelan K. (2021). Dietary fibre in gastrointestinal health and disease. Nat. Rev. Gastroenterol. Hepatol..

[21] Reynolds A., Mann J., Cummings J., Winter N., Mete E., Te Morenga L. (2019). Carbohydrate quality and human health: A series of systematic reviews and meta-analyses. Lancet.

[22] Martinez B., Ortiz R.M. (2017). Thyroid Hormone Regulation and Insulin Resistance: Insights From Animals Naturally Adapted to Fasting. Physiology.

[23] Akasheh R.T., Kroeger C.M., Trepanowski J.F., Gabel K., Hoddy K.K.H., Kalam F., Cienfuegos S., Varady K.A. (2020). Weight loss efficacy of alternate day fasting versus daily calorie restriction in subjects with subclinical hypothyroidism: A secondary analysis. Appl. Physiol. Nutr. Metab..

[24] Fontana L., Klein S., Holloszy J.O., Premachandra B.N. (2006). Effect of long-term calorie restriction with adequate protein and micronutrients on thyroid hormones. J. Clin. Endocrinol. Metab..

[25] Cienfuegos S., Corapi S., Gabel K., Ezpeleta M., Kalam F., Lin S., Pavlou V., Varady K.A. (2022). Effect of Intermittent Fasting on Reproductive Hormone Levels in Females and Males: A Review of Human Trials. Nutrients.

[26] Kalam F., Akasheh R.T., Cienfuegos S., Ankireddy A., Gabel K., Ezpeleta M., Lin S., Tamatam C.M., Reddy S.P., Spring B. (2023). Effect of time-restricted eating on sex hormone levels in premenopausal and postmenopausal females. Obesity.

[27] Liang Y., Yin W., Luo C., Sun L., Feng T., Zhang Y., Yin Y., Zhang W. (2023). Maternal intermittent fasting in mice disrupts the intestinal barrier leading to metabolic disorder in adult offspring. Commun. Biol..

[28] Treasure J., Duarte T.A., Schmidt U. (2020). Eating disorders. Lancet.

[29] Alvarez Ruiz E.M., Gutiérrez-Rojas L. (2015). Comorbidity of bipolar disorder and eating disorders. Rev. Psiquiatr. Salud Ment..

[30] Altman S.E., Shankman S.A. (2009). What is the association between obsessive–compulsive disorder and eating disorders?. Clin. Psychol. Rev..

[31] Barati M., Ghahremani A., Ahmadabad A.H. (2023). Intermittent fasting: A promising dietary intervention for autoimmune diseases. Autoimmun. Rev..

[32] Janssen H., Kahles F., Liu D., Downey J., Koekkoek L.L., Roudko V., D’souza D., McAlpine C.S., Halle L., Poller W.C. (2023). Monocytes re-enter the bone marrow during fasting and alter the host response to infection. Immunity.

[33] Cantoni C., Dorsett Y., Fontana L., Zhou Y., Piccio L. (2022). Effects of dietary restriction on gut microbiota and CNS autoimmunity. Clin. Immunol..

[34] Saglam D., Colak G.A., Sahin E., Ekren B.Y., Sezerman U., Bas M. (2023). Effects of Ramadan intermittent fasting on gut microbiome: Is the diet key?. Front. Microbiol..

[35] Hu X., Xia K., Dai M., Han X., Yuan P., Liu J., Liu S., Jia F., Chen J., Jiang F. (2023). Intermittent fasting modulates the intestinal microbiota and improves obesity and host energy metabolism. NPJ Biofilms Microbiomes.

[36] Mohr A.E., Jasbi P., Bowes D.A., Dirks B., Whisner C.M., Arciero K.M., Poe M., Gu H., Gumpricht E., Sweazea K.L. (2022). Exploratory analysis of one versus two-day intermittent fasting protocols on the gut microbiome and plasma metabolome in adults with overweight/obesity. Front. Nutr..

[37] Khan M.N., Rana M.I., Ayyaz A., Khan M.Y., Imran M. (2022). Intermittent fasting positively modulates human gut microbial diversity and ameliorates blood lipid profile. Front. Microbiol..

[38] Su J., Wang Y., Zhang X., Ma M., Xie Z., Pan Q., Ma Z., Peppelenbosch M.P. (2021). Remodeling of the gut microbiome during Ramadan-associated intermittent fasting. Am. J. Clin. Nutr..

[39] Stanislawski M.A., Frank D.N., Borengasser S.J., Ostendorf D.M., Ir D., Jambal P., Bing K., Wayland L., Siebert J.C., Bessesen D.H. (2021). The Gut Microbiota during a Behavioral Weight Loss Intervention. Nutrients.

[40] Guo Y., Luo S., Ye Y., Yin S., Fan J., Xia M. (2021). Intermittent Fasting Improves Cardiometabolic Risk Factors and Alters Gut Microbiota in Metabolic Syndrome Patients. J. Clin. Endocrinol. Metab..

[41] Mohammadzadeh A., Roshanravan N., Alamdari N.M., Safaiyan A., Mosharkesh E., Hadi A., Barati M., Ostadrahimi A. (2021). The interplay between fasting, gut microbiota, and lipid profile. Int. J. Clin. Pract..

[42] Maifeld A., Bartolomaeus H., Löber U., Avery E.G., Steckhan N., Markó L., Wilck N., Hamad I., Šušnjar U., Mähler A. (2021). Fasting alters the gut microbiome reducing blood pressure and body weight in metabolic syndrome patients. Nat. Commun..

[43] Zouhal H., Bagheri R., Ashtary-Larky D., Wong A., Triki R., Hackney A.C., Laher I., Ben Abderrahman A. (2020). Effects of Ramadan intermittent fasting on inflammatory and biochemical biomarkers in males with obesity. Physiol. Behav..

[44] Gabel K., Marcell J., Cares K., Kalam F., Cienfuegos S., Ezpeleta M., Varady K.A. (2020). Effect of time restricted feeding on the gut microbiome in adults with obesity: A pilot study. Nutr. Health..

[45] Mesnage R., Grundler F., Schwiertz A., Le Maho Y., de Toledo F.W. (2019). Changes in human gut microbiota composition are linked to the energy metabolic switch during 10 d of Buchinger fasting. J. Nutr. Sci..

[46] Ozkul C., Yalinay M., Karakan T. (2019). Islamic fasting leads to an increased abundance of Akkermansia muciniphila and Bacteroides fragilis group: A preliminary study on intermittent fasting. Turk. J. Gastroenterol..

[47] Cignarella F., Cantoni C., Ghezzi L., Salter A., Dorsett Y., Chen L., Phillips D., Weinstock G.M., Fontana L., Cross A.H. (2018). Intermittent Fasting Confers Protection in CNS Autoimmunity by Altering the Gut Microbiota. Cell Metab..

[48] Wang S., Wang J., Zhang J., Liu W., Jing W., Lyu B., Yu H., Zhang Z. (2023). Insoluble Dietary Fiber from Okara Combined with Intermittent Fasting Treatment Synergistically Confers Antiobesity Effects by Regulating Gut Microbiota and Its Metabolites. J. Agric. Food Chem..

[49] Wang J., Zhao X., Zhou R., Wang M., Xiang W., You Z., Li M., Tang R., Zheng J., Li J. (2023). Gut microbiota and transcriptome dynamics in every-other-day fasting are associated with neuroprotection in rats with spinal cord injury. Front. Microbiol..

[50] Teker H.T., Ceylani T. (2023). Intermittent fasting supports the balance of the gut microbiota composition. Int. Microbiol..

[51] Xia J., Guo W., Hu M., Jin X., Zhang S., Liu B., Qiu H., Wang K., Zhuge A., Li S. (2023). Resynchronized rhythmic oscillations of gut microbiota drive time-restricted feeding induced nonalcoholic steatohepatitis alleviation. Gut Microbes.

[52] Ma R.-X., Hu J.-Q., Fu W., Zhong J., Cao C., Wang C.-C., Qi S.-Q., Zhang X.-L., Liu G.-H., Gao Y.-D. (2023). Intermittent fasting protects against food allergy in a murine model via regulating gut microbiota. Front. Immunol..

[53] Su J., Li F., Wang Y., Su Y., Verhaar A., Ma Z., Peppelenbosch M.P. (2022). Investigating Ramadan Like Fasting Effects on the Gut Microbiome in BALB/c Mice. Front. Nutr..

[54] Pan R.-Y., Zhang J., Wang J., Wang Y., Li Z., Liao Y., Liao Y., Zhang C., Liu Z., Song L. (2022). Intermittent fasting protects against Alzheimer’s disease in mice by altering metabolism through remodeling of the gut microbiota. Nat. Aging.

[55] Yang H., Li C., Che M., Li Y., Feng R., Sun C. (2023). Gut microbiota mediates the anti-obesity effect of intermittent fasting by inhibiting intestinal lipid absorption. J. Nutr. Biochem..

[56] Wu J., Man D., Shi D., Wu W., Wang S., Wang K., Li Y., Yang L., Bian X., Wang Q. (2022). Intermittent Fasting Alleviates Risk Markers in a Murine Model of Ulcerative Colitis by Modulating the Gut Microbiome and Metabolome. Nutrients.

[57] Hernandez A.R., Watson C., Federico Q.P., Fletcher R., Brotgandel A., Buford T.W., Carter C.S., Burke S.N. (2022). Twelve Months of Time-Restricted Feeding Improves Cognition and Alters Microbiome Composition Independent of Macronutrient Composition. Nutrients.

[58] Gregor A., Huber L., Auernigg-Haselmaier S., Sternberg F., Billerhart M., Dunkel A., Somoza V., Ogris M., Kofler B., Longo V.D. (2022). A Comparison of the Impact of Restrictive Diets on the Gastrointestinal Tract of Mice. Nutrients.

[59] Xie S., Guan C., Huang T., Liu Y., Yuan F., Xu D. (2022). Intermittent fasting promotes repair of rotator cuff injury in the early postoperative period by regulating the gut microbiota. J. Orthop. Translat..

[60] Shi H., Zhang B., Abo-Hamzy T., Nelson J.W., Ambati C.S.R., Petrosino J.F., Bryan R.M., Durgan D.J. (2021). Restructuring the Gut Microbiota by Intermittent Fasting Lowers Blood Pressure. Circ. Res..

[61] Zhang Z., Chen X., Loh Y.J., Yang X., Zhang C. (2021). The effect of calorie intake, fasting, and dietary composition on metabolic health and gut microbiota in mice. BMC Biol..

[62] Liu J., Zhong Y., Luo X.M., Ma Y., Liu J., Wang H. (2021). Intermittent Fasting Reshapes the Gut Microbiota and Metabolome and Reduces Weight Gain More Effectively Than Melatonin in Mice. Front. Nutr..

[63] Deng Y., Liu W., Wang J., Yu J., Yang L.-Q. (2020). Intermittent Fasting Improves Lipid Metabolism Through Changes in Gut Microbiota in Diet-Induced Obese Mice. Med. Sci. Monit..

[64] Ye Y., Xu H., Xie Z., Wang L., Sun Y., Yang H., Hu D., Mao Y. (2020). Time-Restricted Feeding Reduces the Detrimental Effects of a High-Fat Diet, Possibly by Modulating the Circadian Rhythm of Hepatic Lipid Metabolism and Gut Microbiota. Front. Nutr..

[65] Liu Z., Dai X., Zhang H., Shi R., Hui Y., Jin X., Zhang W., Wang L., Wang Q., Wang D. (2020). Gut microbiota mediates intermittent-fasting alleviation of diabetes-induced cognitive impairment. Nat. Commun..

[66] Li L., Su Y., Li F., Wang Y., Ma Z., Li Z., Su J. (2020). The effects of daily fasting hours on shaping gut microbiota in mice. BMC Microbiol..

[67] Beli E., Yan Y., Moldovan L., Vieira C.P., Gao R., Duan Y., Prasad R., Bhatwadekar A., White F.A., Townsend S.D. (2018). Restructuring of the Gut Microbiome by Intermittent Fasting Prevents Retinopathy and Prolongs Survival in *db/db* Mice. Diabetes.

[68] Agus A., Clément K., Sokol H. (2021). Gut microbiota-derived metabolites as central regulators in metabolic disorders. Gut.

[69] Esteve-Llorens X., Darriba C., Moreira M.T., Feijoo G., González-García S. (2019). Towards an environmentally sustainable and healthy Atlantic dietary pattern: Life cycle carbon footprint and nutritional quality. Sci. Total. Environ..

[70] Krautkramer K.A., Fan J., Bäckhed F. (2021). Gut microbial metabolites as multi-kingdom intermediates. Nat. Rev. Microbiol..

[71] Wei H., Yu C., Zhang C., Ren Y., Guo L., Wang T., Chen F., Li Y., Zhang X., Wang H. (2023). Butyrate ameliorates chronic alcoholic central nervous damage by suppressing microglia-mediated neuroinflammation and modulating the microbiome-gut-brain axis. Biomed. Pharmacother..

[72] Yang Y., Zhong Z., Wang B., Wang Y. (2022). Xiaoyao San ameliorates high-fat diet-induced anxiety and depression via regulating gut microbiota in mice. Biomed. Pharmacother..

[73] Su S., Chen M., Wu Y., Lin Q., Wang D., Sun J., Hai J. (2023). Fecal microbiota transplantation and short-chain fatty acids protected against cognitive dysfunction in a rat model of chronic cerebral hypoperfusion. CNS Neurosci. Ther..

[74] Kong Q., Wang B., Tian P., Li X., Zhao J., Zhang H., Chen W., Wang G. (2021). Daily intake of *Lactobacillus* alleviates autistic-like behaviors by ameliorating the 5-hydroxytryptamine metabolic disorder in VPA-treated rats during weaning and sexual maturation. Food Funct..

[75] Tu R., Xia J. (2023). Stroke and Vascular Cognitive Impairment: The Role of Intestinal Microbiota Metabolite TMAO. CNS Neurol. Disord. Drug Targets.

[76] Zhong C., Lu Z., Che B., Qian S., Zheng X., Wang A., Bu X., Zhang J., Ju Z., Xu T. (2021). Choline Pathway Nutrients and Metabolites and Cognitive Impairment After Acute Ischemic Stroke. Stroke.

[77] MahmoudianDehkordi S., Arnold M., Nho K., Ahmad S., Jia W., Xie G., Louie G., Kueider-Paisley A., Moseley M.A., Thompson J.W. (2019). Altered bile acid profile associates with cognitive impairment in Alzheimer’s disease—An emerging role for gut microbiome. Alzheimer’s Dement..

[78] Nie K., Li Y., Zhang J., Gao Y., Qiu Y., Gan R., Zhang Y., Wang L. (2022). Distinct Bile Acid Signature in Parkinson’s Disease With Mild Cognitive Impairment. Front. Neurol..

[79] Wang X., Sun G., Feng T., Zhang J., Huang X., Wang T., Xie Z., Chu X., Yang J., Wang H. (2019). Sodium oligomannate therapeutically remodels gut microbiota and suppresses gut bacterial amino acids-shaped neuroinflammation to inhibit Alzheimer’s disease progression. Cell Res..

[80] Sun P., Wang M., Li Z., Wei J., Liu F., Zheng W., Zhu X., Chai X., Zhao S. (2022). *Eucommiae cortex* polysaccharides mitigate obesogenic diet-induced cognitive and social dysfunction via modulation of gut microbiota and tryptophan metabolism. Theranostics.

[81] Camacho-Barcia L., García-Gavilán J., Martínez-González M., Fernández-Aranda F., Galié S., Corella D., Cuenca-Royo A., Romaguera D., Vioque J., Alonso-Gómez M. (2022). Vitamin K dietary intake is associated with cognitive function in an older adult Mediterranean population. Age Ageing.

[82] Ford A.H., Almeida O.P. (2019). Effect of Vitamin B Supplementation on Cognitive Function in the Elderly: A Systematic Review and Meta-Analysis. Drugs Aging.

[83] Tan J.K., Macia L., Mackay C.R. (2023). Dietary fiber and SCFAs in the regulation of mucosal immunity. J. Allergy Clin. Immunol..

[84] Dalile B., Van Oudenhove L., Vervliet B., Verbeke K. (2019). The role of short-chain fatty acids in microbiota–gut–brain communication. Nat. Rev. Gastroenterol. Hepatol..

[85] Kim C.H. (2021). Control of lymphocyte functions by gut microbiota-derived short-chain fatty acids. Cell. Mol. Immunol..

[86] Hu T., Wu Q., Yao Q., Jiang K., Yu J., Tang Q. (2022). Short-chain fatty acid metabolism and multiple effects on cardiovascular diseases. Ageing Res. Rev..

[87] He P., Yu L., Tian F., Zhang H., Chen W., Zhai Q. (2022). Dietary Patterns and Gut Microbiota: The Crucial Actors in Inflammatory Bowel Disease. Adv. Nutr..

[88] Yang J., Yu J. (2018). The association of diet, gut microbiota and colorectal cancer: What we eat may imply what we get. Protein Cell.

[89] Canfora E.E., Jocken J.W., Blaak E.E. (2015). Short-chain fatty acids in control of body weight and insulin sensitivity. Nat. Rev. Endocrinol..

[90] Yang B., Xiong Z., Lin M., Yang Y., Chen Y., Zeng J., Jia X., Feng L. (2023). Astragalus polysaccharides alleviate type 1 diabetes via modulating gut microbiota in mice. Int. J. Biol. Macromol..

[91] May K.S., Hartigh L.J.D. (2021). Modulation of Adipocyte Metabolism by Microbial Short-Chain Fatty Acids. Nutrients.

[92] Pluznick J.L., Protzko R.J., Gevorgyan H., Peterlin Z., Sipos A., Han J., Brunet I., Wan L.X., Rey F., Wang T. (2013). Olfactory receptor responding to gut microbiota derived signals plays a role in renin secretion and blood pressure regulation. Proc. Natl. Acad. Sci. USA.

[93] Tang C.-F., Wang C.-Y., Wang J.-H., Wang Q.-N., Li S.-J., Wang H.-O., Zhou F., Li J.-M. (2022). Short-Chain Fatty Acids Ameliorate Depressive-like Behaviors of High Fructose-Fed Mice by Rescuing Hippocampal Neurogenesis Decline and Blood–Brain Barrier Damage. Nutrients.

[94] Doroszkiewicz J., Groblewska M., Mroczko B. (2021). The Role of Gut Microbiota and Gut–Brain Interplay in Selected Diseases of the Central Nervous System. Int. J. Mol. Sci..

[95] Song L., Sun Q., Zheng H., Zhang Y., Wang Y., Liu S., Duan L. (2022). *Roseburia hominis* Alleviates Neuroinflammation via Short-Chain Fatty Acids through Histone Deacetylase Inhibition. Mol. Nutr. Food Res..

[96] Qi Q., Li J., Yu B., Moon J.-Y., Chai J.C., Merino J., Hu J., Ruiz-Canela M., Rebholz C., Wang Z. (2021). Host and gut microbial tryptophan metabolism and type 2 diabetes: An integrative analysis of host genetics, diet, gut microbiome and circulating metabolites in cohort studies. Gut.

[97] Arnoriaga-Rodríguez M., Mayneris-Perxachs J., Contreras-Rodríguez O., Burokas A., Ortega-Sanchez J.-A., Blasco G., Coll C., Biarnés C., Castells-Nobau A., Puig J. (2021). Obesity-associated deficits in inhibitory control are phenocopied to mice through gut microbiota changes in one-carbon and aromatic amino acids metabolic pathways. Gut.

[98] Pascual F., Camilli S., Lockey R.F., Kolliputi N. (2023). Mind-body connection: Metabolite 4-ethylphenyl linked to anxiety behavior and oligodendrocyte modification in autism spectrum disorder. Am. J. Physiol. Gastrointest. Liver Physiol..

[99] Niu H., Zhou X., Gong P., Jiao Y., Zhang J., Wu Y., Lyu L., Liang C., Chen S., Han X. (2022). Effect of *Lactobacillus rhamnosus* MN-431 Producing Indole Derivatives on Complementary Feeding-Induced Diarrhea Rat Pups Through the Enhancement of the Intestinal Barrier Function. Mol. Nutr. Food Res..

[100] Chimerel C., Emery E., Summers D.K., Keyser U., Gribble F.M., Reimann F. (2014). Bacterial metabolite indole modulates incretin secretion from intestinal enteroendocrine L cells. Cell Rep..

[101] Targher G., Mantovani A., Byrne C.D. (2023). Mechanisms and possible hepatoprotective effects of glucagon-like peptide-1 receptor agonists and other incretin receptor agonists in non-alcoholic fatty liver disease. Lancet Gastroenterol. Hepatol..

[102] Cervenka I., Agudelo L.Z., Ruas J.L. (2017). Kynurenines: Tryptophan’s metabolites in exercise, inflammation, and mental health. Science.

[103] Agudelo L.Z., Femenía T., Orhan F., Porsmyr-Palmertz M., Goiny M., Martinez-Redondo V., Correia J.C., Izadi M., Bhat M., Schuppe-Koistinen I. (2014). Skeletal muscle PGC-1α1 modulates kynurenine metabolism and mediates resilience to stress-induced depression. Cell.

[104] Mayneris-Perxachs J., Castells-Nobau A., Arnoriaga-Rodríguez M., Martin M., de la Vega-Correa L., Zapata C., Burokas A., Blasco G., Coll C., Escrichs A. (2022). Microbiota alterations in proline metabolism impact depression. Cell Metab..

[105] Ritter C., Buchmann A., Müller S.T., Volleberg M., Haynes M., Ghisleni C., Noeske R., Tuura R., Hasler G. (2022). Evaluation of Prefrontal γ-Aminobutyric Acid and Glutamate Levels in Individuals With Major Depressive Disorder Using Proton Magnetic Resonance Spectroscopy. JAMA Psychiatry.

[106] Xia H., Chen H., Cheng X., Yin M., Yao X., Ma J., Huang M., Chen G., Liu H. (2022). Zebrafish: An efficient vertebrate model for understanding role of gut microbiota. Mol. Med..

[107] Fiaschini N., Mancuso M., Tanori M., Colantoni E., Vitali R., Diretto G., Rebenaque L.L., Stronati L., Negroni A. (2022). Liver Steatosis and Steatohepatitis Alter Bile Acid Receptors in Brain and Induce Neuroinflammation: A Contribution of Circulating Bile Acids and Blood-Brain Barrier. Int. J. Mol. Sci..

[108] Guan B., Tong J., Hao H., Yang Z., Chen K., Xu H., Wang A. (2022). Bile acid coordinates microbiota homeostasis and systemic immunometabolism in cardiometabolic diseases. Acta Pharm. Sin. B.

[109] Ren Z.-L., Li C.-X., Ma C.-Y., Chen D., Chen J.-H., Xu W.-X., Chen C.-A., Cheng F.-F., Wang X.-Q. (2022). Linking Nonalcoholic Fatty Liver Disease and Brain Disease: Focusing on Bile Acid Signaling. Int. J. Mol. Sci..

[110] Peterson L.W., Artis D. (2014). Intestinal epithelial cells: Regulators of barrier function and immune homeostasis. Nat. Rev. Immunol..

[111] Horowitz A., Chanez-Paredes S.D., Haest X., Turner J.R. (2023). Paracellular permeability and tight junction regulation in gut health and disease. Nat. Rev. Gastroenterol. Hepatol..

[112] Zhao Z., Li F., Ning J., Peng R., Shang J., Liu H., Shang M., Bao X.-Q., Zhang D. (2021). Novel compound FLZ alleviates rotenone-induced PD mouse model by suppressing TLR4/MyD88/NF-κB pathway through microbiota–gut–brain axis. Acta Pharm. Sin. B.

[113] Hu R., Li S., Diao H., Huang C., Yan J., Wei X., Zhou M., He P., Wang T., Fu H. (2023). The interaction between dietary fiber and gut microbiota, and its effect on pig intestinal health. Front. Immunol..

[114] Berthoud H.-R., Albaugh V.L., Neuhuber W.L. (2021). Gut-brain communication and obesity: Understanding functions of the vagus nerve. J. Clin. Investig..

[115] Prescott S.L., Liberles S.D. (2022). Internal senses of the vagus nerve. Neuron.

[116] Strader A.D., Woods S.C. (2005). Gastrointestinal hormones and food intake. Gastroenterology.

[117] Bravo J.A., Forsythe P., Chew M.V., Escaravage E., Savignac H.M., Dinan T.G., Bienenstock J., Cryan J.F. (2011). Ingestion of Lactobacillus strain regulates emotional behavior and central GABA receptor expression in a mouse via the vagus nerve. Proc. Natl. Acad. Sci. USA.

[118] Sgritta M., Dooling S.W., Buffington S.A., Momin E.N., Francis M.B., Britton R.A., Costa-Mattioli M. (2019). Mechanisms Underlying Microbial-Mediated Changes in Social Behavior in Mouse Models of Autism Spectrum Disorder. Neuron.

[119] Deschasaux M., Bouter K.E., Prodan A., Levin E., Groen A.K., Herrema H., Tremaroli V., Bakker G.J., Attaye I., Pinto-Sietsma S.-J. (2018). Depicting the composition of gut microbiota in a population with varied ethnic origins but shared geography. Nat. Med..

[120] Harach T., Marungruang N., Duthilleul N., Cheatham V., Mc Coy K.D., Frisoni G.B., Neher J.J., Fåk F., Jucker M., Lasser T. (2017). Reduction of Abeta amyloid pathology in APPPS1 transgenic mice in the absence of gut microbiota. Sci. Rep..

[121] Minter M.R., Zhang C., Leone V., Ringus D.L., Zhang X., Oyler-Castrillo P., Musch M.W., Liao F., Ward J.F., Holtzman D.M. (2016). Antibiotic-induced perturbations in gut microbial diversity influences neuro-inflammation and amyloidosis in a murine model of Alzheimer’s disease. Sci. Rep..

[122] Vogt N.M., Kerby R.L., Dill-McFarland K.A., Harding S.J., Merluzzi A.P., Johnson S.C., Carlsson C.M., Asthana S., Zetterberg H., Blennow K. (2017). Gut microbiome alterations in Alzheimer’s disease. Sci. Rep..

[123] Bekkering P., Jafri I., van Overveld F.J., Rijkers G.T. (2013). The intricate association between gut microbiota and development of type 1, type 2 and type 3 diabetes. Expert Rev. Clin. Immunol..

[124] Zhao Y., Dua P., Lukiw W.J. (2015). Microbial Sources of Amyloid and Relevance to Amyloidogenesis and Alzheimer’s Disease (AD). J. Alzheimer’s Dis. Parkinsonism..

[125] Bhattacharjee S., Lukiw W.J. (2013). Alzheimer’s disease and the microbiome. Front. Cell. Neurosci..

[126] Syed A.K., Boles B.R. (2014). Fold modulating function: Bacterial toxins to functional amyloids. Front. Microbiol..

[127] Hufnagel D.A., Tükel C., Chapman M.R. (2013). Disease to dirt: The biology of microbial amyloids. PLoS Pathog..

[128] Schwartz K., Boles B.R. (2013). Microbial amyloids—Functions and interactions within the host. Curr. Opin. Microbiol..

[129] Oli M.W., Otoo H.N., Crowley P.J., Heim K.P., Nascimento M.M., Ramsook C.B., Lipke P.N., Brady L.J. (2012). Functional amyloid formation by Streptococcus mutans. Microbiology.

[130] Friedland R.P. (2015). Mechanisms of molecular mimicry involving the microbiota in neurodegeneration. J. Alzheimer’s Dis..

[131] Scott K.P., Gratz S.W., Sheridan P.O., Flint H.J., Duncan S.H. (2013). The influence of diet on the gut microbiota. Pharmacol. Res..

[132] Soto C. (2012). Transmissible Proteins: Expanding the prion heresy. Cell.

[133] Berti V., Murray J., Davies M., Spector N., Tsui W.H., Li Y., Williams S., Pirraglia E., Vallabhajosula S., McHugh P. (2015). Nutrient patterns and brain biomarkers of Alzheimer’s disease in cognitively normal individuals. J. Nutr. Health Aging.

[134] Stefani M., Rigacci S. (2014). Beneficial properties of natural phenols: Highlight on protection against pathological conditions associated with amyloid aggregation. BioFactors.

[135] Rigacci S., Stefani M. (2014). Nutraceuticals and amyloid neurodegenerative diseases: A focus on natural phenols. Expert Rev. Neurother..

[136] Pistollato F., Sumalla Cano S., Elio I., Masias Vergara M., Giampieri F., Battino M. (2016). Role of gut microbiota and nutrients in amyloid formation and pathogenesis of Alzheimer disease. Nutr. Rev..

[137] Lazic D., Tesic V., Jovanovic M., Brkic M., Milanovic D., Zlokovic B.V., Kanazir S., Perovic M. (2020). Every-other-day feeding exacerbates inflammation and neuronal deficits in 5XFAD mouse model of Alzheimer’s disease. Neurobiol. Dis..

[138] Ntsapi C.M., Loos B. (2021). Neurons die with heightened but functional macro- and chaperone mediated autophagy upon increased amyloid-ß induced toxicity with region-specific protection in prolonged intermittent fasting. Exp. Cell Res..

[139] Li W., Wu M., Zhang Y., Wei X., Zang J., Liu Y., Wang Y., Gong C., Wei W. (2020). Intermittent fasting promotes adult hippocampal neuronal differentiation by activating GSK-3β in 3xTg-AD mice. J. Neurochem..

[140] Elesawy B.H., Raafat B.M., Al Muqbali A., Abbas A.M., Sakr H.F. (2021). The Impact of Intermittent Fasting on Brain-Derived Neurotrophic Factor, Neurotrophin 3, and Rat Behavior in a Rat Model of Type 2 Diabetes Mellitus. Brain Sci..

[141] Zhang J., Zhan Z., Li X., Xing A., Jiang C., Chen Y., Shi W., An L. (2017). Intermittent Fasting Protects against Alzheimer’s Disease Possible through Restoring Aquaporin-4 Polarity. Front. Mol. Neurosci..

[142] Halagappa V.K., Guo Z., Pearson M., Matsuoka Y., Cutler R.G., LaFerla F.M., Mattson M.P. (2007). Intermittent fasting and caloric restriction ameliorate age-related behavioral deficits in the triple-transgenic mouse model of Alzheimer’s disease. Neurobiol. Dis..

[143] Park S., Shin B.K. (2022). Intermittent fasting with a high-protein diet mitigated osteoarthritis symptoms by increasing lean body mass and reducing inflammation in osteoarthritic rats with Alzheimer’s disease-like dementia. Br. J. Nutr..

[144] Shin B.K., Kang S., Kim D.S., Park S. (2018). Intermittent fasting protects against the deterioration of cognitive function, energy metabolism and dyslipidemia in Alzheimer’s disease-induced estrogen deficient rats. Exp. Biol. Med..

[145] Tatulli G., Mitro N., Cannata S.M., Audano M., Caruso D., D’arcangelo G., Lettieri-Barbato D., Aquilano K. (2018). Intermittent Fasting Applied in Combination with Rotenone Treatment Exacerbates Dopamine Neurons Degeneration in Mice. Front. Cell. Neurosci..

[146] Ojha U., Khanal S., Park P.-H., Hong J.T., Choi D.-Y. (2023). Intermittent fasting protects the nigral dopaminergic neurons from MPTP-mediated dopaminergic neuronal injury in mice. J. Nutr. Biochem..

[147] Ehrnhoefer D.E., Martin D.D.O., Schmidt M.E., Qiu X., Ladha S., Caron N.S., Skotte N.H., Nguyen Y.T.N., Vaid K., Southwell A.L. (2018). Preventing mutant huntingtin proteolysis and intermittent fasting promote autophagy in models of Huntington disease. Acta Neuropathol. Commun..

[148] Saadatnia M., Etemadifar M., Fatehi F., Ashtari F., Shaygannejad V., Chitsaz A., Maghzi A.H. (2009). Short-term effects of prolonged fasting on multiple sclerosis. Eur. Neurol..

[149] Wingo B.C., Rinker J.R., Green K., Peterson C.M. (2022). Feasibility and acceptability of time-restricted eating in a group of adults with multiple sclerosis. Front. Neurol..

[150] Bai M., Wang Y., Han R., Xu L., Huang M., Zhao J., Lin Y., Song S., Chen Y. (2021). Intermittent caloric restriction with a modified fasting-mimicking diet ameliorates autoimmunity and promotes recovery in a mouse model of multiple sclerosis. J. Nutr. Biochem..

[151] Au A., Feher A., McPhee L., Jessa A., Oh S., Einstein G. (2016). Estrogens, inflammation and cognition. Front. Neuroendocrinol..

[152] Brettschneider J., Del Tredici K., Lee V.M.-Y., Trojanowski J.Q. (2015). Spreading of pathology in neurodegenerative diseases: A focus on human studies. Nat. Rev. Neurosci..

[153] Luk K.C., Kehm V., Carroll J., Zhang B., O’Brien P., Trojanowski J.Q., Lee V.M.-Y. (2012). Pathological α-synuclein transmission initiates parkinson-like neurodegeneration in nontransgenic mice. Science.

[154] Cersosimo M.G., Benarroch E.E. (2012). Autonomic involvement in Parkinson’s disease: Pathology, pathophysiology, clinical features and possible peripheral biomarkers. J. Neurol. Sci..

[155] Cersosimo M.G., Benarroch E.E. (2008). Neural control of the gastrointestinal tract: Implications for Parkinson disease. Mov. Disord..

[156] Pfeiffer R.F. (2018). Gastrointestinal Dysfunction in Parkinson’s Disease. Curr. Treat. Options Neurol..

[157] Braak H., de Vos R.A., Bohl J., Del Tredici K. (2006). Gastric α-synuclein immunoreactive inclusions in Meissner’s and Auerbach’s plexuses in cases staged for Parkinson’s disease-related brain pathology. Neurosci. Lett..

[158] Sampson T.R., Debelius J.W., Thron T., Janssen S., Shastri G.G., Ilhan Z.E., Challis C., Schretter C.E., Rocha S., Gradinaru V. (2016). Gut Microbiota Regulate Motor Deficits and Neuroinflammation in a Model of Parkinson’s Disease. Cell.

[159] Maetzler W., Liepelt I., Berg D. (2009). Progression of Parkinson’s disease in the clinical phase: Potential markers. Lancet Neurol..

[160] Jellinger K.A. (2011). Synuclein deposition and non-motor symptoms in Parkinson disease. J. Neurol. Sci..

[161] Berryman D.E., Glad C.A.M., List E.O., Johannsson G. (2013). The GH/IGF-1 axis in obesity: Pathophysiology and therapeutic considerations. Nat. Rev. Endocrinol..

[162] Godau J., Knauel K., Weber K., Brockmann K., Maetzler W., Binder G., Berg D. (2011). Serum insulinlike growth factor 1 as possible marker for risk and early diagnosis of parkinson disease. Arch. Neurol..

[163] De Felice F.G. (2013). Alzheimer’s disease and insulin resistance: Translating basic science into clinical applications. J. Clin. Investig..

[164] Mattson M.P., Wan R. (2005). Beneficial effects of intermittent fasting and caloric restriction on the cardiovascular and cerebrovascular systems. J. Nutr. Biochem..

[165] Shaw C.S., Clark J., Wagenmakers A.J. (2010). The effect of exercise and nutrition on intramuscular fat metabolism and insulin sensitivity. Annu. Rev. Nutr..

[166] Bishop-Bailey D. (2013). Mechanisms governing the health and performance benefits of exercise. Br. J. Pharmacol..

[167] Kurth T., Moore S.C., Gaziano J.M., Kase C.S., Stampfer M.J., Berger K., Buring J.E. (2006). healthy lifestyle and the risk of stroke in women. Arch. Intern. Med..

[168] Cotman C.W., Berchtold N.C. (2002). Exercise: A behavioral intervention to enhance brain health and plasticity. Trends Neurosci..

[169] Voss M.W., Vivar C., Kramer A.F., van Praag H. (2013). Bridging animal and human models of exercise-induced brain plasticity. Trends Cogn. Sci..

[170] van Praag H. (2008). Neurogenesis and exercise: Past and future directions. NeuroMolecular Med..

[171] Mattson M.P. (2014). Interventions that improve body and brain bioenergetics for Parkinson’s disease risk reduction and therapy. J. Park. Dis..

[172] Walker F.O. (2007). Huntington’s disease. Lancet.

[173] Phillips M.C.L., McManus E.J., Brinkhuis M., Romero-Ferrando B. (2022). Time-Restricted Ketogenic Diet in Huntington’s Disease: A Case Study. Front. Behav. Neurosci..

[174] Phillips M.C. (2022). Metabolic Strategies in Healthcare: A New Era. Aging Dis..

[175] Whittaker D.S., Loh D.H., Wang H.-B., Tahara Y., Kuljis D., Cutler T., Ghiani C.A., Shibata S., Block G.D., Colwell C.S. (2018). Circadian-based Treatment Strategy Effective in the BACHD Mouse Model of Huntington’s Disease. J. Biol. Rhythm..

[176] Wang H.-B., Loh D.H., Whittaker D.S., Cutler T., Howland D., Colwell C.S. (2018). Time-Restricted Feeding Improves Circadian Dysfunction as well as Motor Symptoms in the Q175 Mouse Model of Huntington’s Disease. eNeuro.

[177] McCourt A.C., O’Donovan K.L., Ekblad E., Sand E., Craufurd D., Rosser A., Sanders D., Stoy N., Rickards H., Wierup N. (2015). Characterization of Gastric Mucosa Biopsies Reveals Alterations in Huntington’s Disease. PLoS Curr..

[178] Moffitt H., McPhail G.D., Woodman B., Hobbs C., Bates G.P. (2009). Formation of polyglutamine inclusions in a wide range of non-CNS tissues in the HdhQ150 knock-in mouse model of huntington’s disease. PLoS ONE.

[179] Dendrou C.A., Fugger L., Friese M.A. (2015). Immunopathology of multiple sclerosis. Nat. Rev. Immunol..

[180] Steinman L., Zamvil S.S. (2005). Virtues and pitfalls of EAE for the development of therapies for multiple sclerosis. Trends Immunol..

[181] Wang Y., Fu Z., Li X., Liang Y., Pei S., Hao S., Zhu Q., Yu T., Pei Y., Yuan J. (2021). Cytoplasmic DNA sensing by KU complex in aged CD4(+) T cell potentiates T cell activation and aging-related autoimmune inflammation. Immunity.

[182] Carbone F., La Rocca C., De Candia P., Procaccini C., Colamatteo A., Micillo T., De Rosa V., Matarese G. (2016). Metabolic control of immune tolerance in health and autoimmunity. Semin. Immunol..

[183] Calder P.C., Ahluwalia N., Brouns F., Buetler T., Clement K., Cunningham K., Esposito K., Jönsson L.S., Kolb H., Lansink M. (2011). Dietary factors and low-grade inflammation in relation to overweight and obesity. Br. J. Nutr..

[184] Berer K., Mues M., Koutrolos M., Al Rasbi Z., Boziki M., Johner C., Wekerle H., Krishnamoorthy G. (2011). Commensal microbiota and myelin autoantigen cooperate to trigger autoimmune demyelination. Nature.

[185] Lee Y.K., Menezes J.S., Umesaki Y., Mazmanian S.K. (2011). Proinflammatory T-cell responses to gut microbiota promote experimental autoimmune encephalomyelitis. Proc. Natl. Acad. Sci. USA.

[186] Ochoa-Repáraz J., Mielcarz D.W., Ditrio L.E., Burroughs A.R., Foureau D.M., Haque-Begum S., Kasper L.H. (2009). Role of gut commensal microflora in the development of experimental autoimmune encephalomyelitis. J. Immunol..

[187] Vieira S.M., Pagovich O.E., Kriegel M.A. (2014). Diet, microbiota and autoimmune diseases. Lupus.

[188] Wu H.-J., Ivanov I.I., Darce J., Hattori K., Shima T., Umesaki Y., Littman D.R., Benoist C., Mathis D. (2010). Gut-residing segmented filamentous bacteria drive autoimmune arthritis via T helper 17 cells. Immunity.

[189] Jangi S., Gandhi R., Cox L.M., Li N., von Glehn F., Yan R., Patel B., Mazzola M.A., Liu S., Glanz B.L. (2016). Alterations of the human gut microbiome in multiple sclerosis. Nat. Commun..

[190] Piccio L., Stark J.L., Cross A.H. (2008). Chronic calorie restriction attenuates experimental autoimmune encephalomyelitis. J. Leukoc. Biol..

[191] Henninger N., Kumar R., Fisher M. (2010). Acute ischemic stroke therapy. Expert Rev. Cardiovasc. Ther..

[192] Iadecola C., Anrather J. (2011). The immunology of stroke: From mechanisms to translation. Nat. Med..

[193] Mazmanian S.K., Liu C.H., Tzianabos A.O., Kasper D.L. (2005). An immunomodulatory molecule of symbiotic bacteria directs maturation of the host immune system. Cell.

[194] Benakis C., Brea D., Caballero S., Faraco G., Moore J., Murphy M., Sita G., Racchumi G., Ling L., Pamer E.G. (2016). Commensal microbiota affects ischemic stroke outcome by regulating intestinal γδ T cells. Nat. Med..

[195] Prinz I., Silva-Santos B., Pennington D.J. (2013). Functional development of γδ T cells. Eur. J. Immunol..

[196] Gelderblom M., Weymar A., Bernreuther C., Velden J., Arunachalam P., Steinbach K., Orthey E., Arumugam T.V., Leypoldt F., Simova O. (2012). Neutralization of the IL-17 axis diminishes neutrophil invasion and protects from ischemic stroke. Blood.

[197] Iadecola C., Buckwalter M.S., Anrather J. (2020). Immune responses to stroke: Mechanisms, modulation, and therapeutic potential. J. Clin. Investig..

[198] Fann D.Y.-W., Santro T., Manzanero S., Widiapradja A., Cheng Y.-L., Lee S.-Y., Chunduri P., Jo D.-G., Stranahan A.M., Mattson M.P. (2014). Intermittent fasting attenuates inflammasome activity in ischemic stroke. Exp. Neurol..

[199] Magdy R., Kishk N.A., Abokrysha N.T., Ramzy G.M., Rizk H.I., Hussein M. (2022). Fasting and post fasting effect of Ramadan on different seizure types in patients with active epilepsy. Nutr. Neurosci..

[200] Luo A., Yan J., Tang X., Zhao Y., Zhou B., Li S. (2019). Postoperative cognitive dysfunction in the aged: The collision of neuroinflammaging with perioperative neuroinflammation. Inflammopharmacology.

[201] Scott D.A., Evered L.A. (2019). Caring for the ageing mind. Anaesthesia.

[202] Jiang X., Gu X., Zhou X., Chen X., Zhang X., Yang Y., Qin Y., Shen L., Yu W., Su D. (2019). Intestinal dysbacteriosis mediates the reference memory deficit induced by anaesthesia/surgery in aged mice. Brain Behav. Immun..

[203] Marcucci M., Chan M.T.V., Smith E.E., Absalom A.R., Devereaux P.J. (2023). Prevention of perioperative stroke in patients undergoing non-cardiac surgery. Lancet Neurol..

[204] Bi J., Xu Y., Li S., Zhan G., Hua D., Tan J., Chi X., Xiang H., Guo F., Luo A. (2023). Contribution of preoperative gut microbiota in postoperative neurocognitive dysfunction in elderly patients undergoing orthopedic surgery. Front. Aging Neurosci..

[205] Hua D., Li S., Li S., Wang X., Wang Y., Xie Z., Zhao Y., Zhang J., Luo A. (2021). Gut Microbiome and Plasma Metabolome Signatures in Middle-Aged Mice With Cognitive Dysfunction Induced by Chronic Neuropathic Pain. Front. Mol. Neurosci..

[206] Abdelhamid Y.A., Chapman M.J., Deane A.M. (2016). Peri-operative nutrition. Anaesthesia.

[207] Huang W., Yan Y., Wu M., Hu J., Zhao J., Chen X., Liu W., Liu K., Li C. (2022). Preoperative fasting confers protection against intestinal ischaemia/reperfusion injury by modulating gut microbiota and their metabolites in a mouse model. Br. J. Anaesth..

[208] Sun R., Zhou Z., Li X., Xu Q., Zhou B., Yu H., Zhang W., Sun Q., Zhang X., Luo X. (2023). Prognostic significance of preoperative nutritional status for postoperative acute kidney injury in older patients undergoing major abdominal surgery: A retrospective cohort study. Int. J. Surg..

